# Influence of hidden halogen mobility on local structure of CsSn(Cl_1−*x*_Br_*x*_)_3_ mixed-halide perovskites by solid-state NMR†

**DOI:** 10.1039/d0sc05614f

**Published:** 2020-12-30

**Authors:** Abhoy Karmakar, Amit Bhattacharya, Diganta Sarkar, Guy M. Bernard, Arthur Mar, Vladimir K. Michaelis

**Affiliations:** Department of Chemistry, University of Alberta Edmonton Alberta T6G 2G2 Canada vladimir.michaelis@ualberta.ca

## Abstract

Tin halide perovskites are promising candidates for lead-free photovoltaic and optoelectronic materials, but not all of them have been well characterized. It is essential to determine how the bulk photophysical properties are correlated with their structures at both short and long ranges. Although CsSnCl_3_ is normally stable in the cubic perovskite structure only above 379 K, it was prepared as a metastable phase at room temperature. The transition from the cubic to the monoclinic phase, which is the stable form at room temperature, was tracked by solid-state ^133^Cs NMR spectroscopy and shown to take place through a first-order kinetics process. The complete solid solution CsSn(Cl_1−*x*_Br_*x*_)_3_ (0 ≤ *x* ≤ 1) was successfully prepared, exhibiting cubic perovskite structures extending between the metastable CsSnCl_3_ and stable CsSnBr_3_ end-members. The NMR spectra of CsSnBr_3_ samples obtained by three routes (high-temperature, mechanochemical, and solvent-assisted reactions) show distinct chemical shift ranges, spin-lattice relaxation parameters and peak widths, indicative of differences in local structure, defects and degree of crystallinity within these samples. Variable-temperature ^119^Sn spin-lattice relaxation measurements reveal spontaneous mobility of Br atoms in CsSnBr_3_. The degradation of CsSnBr_3_, exposed to an ambient atmosphere for nearly a year, was monitored by NMR spectroscopy and powder X-ray diffraction, as well as by optical absorption spectroscopy.

## Abstract 2

Unravelling the atomic-level chemical structure, slow phase conversion or degradation pathways and rapid halogen hopping of cesium tin(ii) halide perovskites using solid-state ^119^Sn and ^133^Cs NMR spectroscopy.

## Graphical abstract

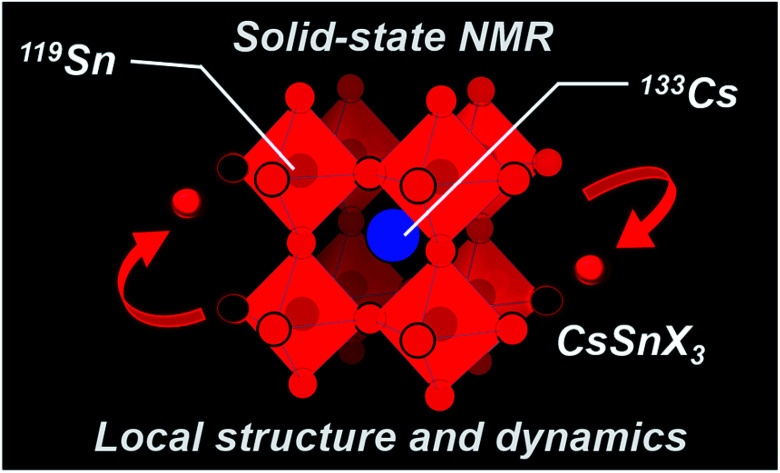

## 1. Introduction

Lead halide perovskites APbX_3_ (A = Cs^+^, CH_3_NH_3_^+^, CH(NH_2_)_2_^+^; X = Cl^−^, Br^−^, I^−^) show attractive optical and electrical properties for solar cells, lasers, light-emitting diodes, X-ray detectors, and other applications,^1–6^ with power conversion efficiencies now exceeding 25%,^7^ but they face significant challenges for commercialization because of poor chemical stability^8^ and risk of lead toxicity.^9,10^ Among lead-free alternatives, the tin-substituted analogues have been highly promising, showing power conversion efficiencies of up to 10%.^11^ Most of these compounds are iodides such as CH_3_NH_3_SnI_3_, CH(NH_2_)_2_SnI_3_, and CsSnI_3_, which have band gaps (1.2–1.4 eV) close to the optimum value (1.34 eV) to maximize efficiency.^12,13^ The hybrid organic–inorganic compounds CH_3_NH_3_SnI_3_ and CH(NH_2_)_2_SnI_3_ are more efficient but suffer from poor thermal stability because of the presence of the volatile organic cations; the purely inorganic compound CsSnI_3_ is more thermally stable but oxidizes rapidly in air (Sn(+2) → Sn(+4)).^13,14^ Among the bromide analogues, CsSnBr_3_ exhibits greater thermal and air stability, imparted by the Cs^+^, than CH_3_NH_3_SnBr_3_, which degrades in air within an hour.^13,15^ Solar devices fabricated with CsSnBr_3_ do not have to be encapsulated, lasting for hours with diode characteristics being retained.^13^ The band gap of CsSnBr_3_ (1.75–1.80 eV)^13,16,17^ lies in the optimal region for tandem solar cell technology.^18^

Recently, the mixed-halide perovskites CsSn(Cl_1–*x*_Br_*x*_)_3_ have been prepared in the form of nanocrystals or thin films, in which the band gap can be adjusted with composition, making them suitable for optoelectronic applications such as light emitting diodes and lasers.^19,20^ However, some of the structural details of these mixed-halide perovskites are unclear because the end-members are known to exhibit multiple phase transitions: CsSnCl_3_ adopts a monoclinic structure at room temperature (own type; space group originally reported as *P*2_1_/*n*, but standardized as *P*2_1_/*c*) and transforms to the cubic perovskite structure (space group *Pm*3̄*m*) above 379 K,^21^ and CsSnBr_3_ undergoes complicated phase transitions at low temperature, but ultimately attains the cubic structure above 292 K.^22^ Because the physical properties depend sensitively on the structure and stability of these mixed-halide perovskites, it is essential to determine the local and long-range atomic arrangement, to unravel the dynamics of halogen mobility, and to evaluate changes entailed by different synthetic methods and exposure to ambient conditions.

To date, halide perovskites have been extensively characterized by X-ray diffraction (XRD), which gives information about the average long-range structure. However, solid-state nuclear magnetic resonance (NMR) spectroscopy is an invaluable method to investigate local structure (*e.g.*, halide distribution, domain structure) and dynamics in perovskites.^23–38^ In particular, ^133^Cs (*I* = 7/2, 100% abundance) and ^119^Sn (*I* = 1/2, 8.59% abundance) are ideal NMR-sensitive nuclei, useful for probing the local structure of the A and B sites in perovskites ABX_3_,^30,37,39–45^ as well as of sites in other types of compounds.^46–49^

Here we target the preparation of CsSn(Cl_1−*x*_Br_*x*_)_3_ to ascertain if a complete solid solution with the cubic perovskite structure can be attained for the entire range. Powder XRD and NMR spectroscopy were carried out to determine the long-range structure and the local coordination around the Cs and Sn sites by Cl and Br atoms, which could be ordered or disordered. Optical band gaps were measured by UV-visible absorption spectroscopy and correlated with composition. Given the ambiguity about the nature of CsSnBr_3_, we evaluate whether samples prepared by various methods show important structural differences which could influence their optical properties. The activation energy for rapid halogen dynamics in CsSnBr_3_ was quantified by variable-temperature ^119^Sn NMR spectroscopy. Finally, the stability of CsSnBr_3_ under ambient conditions was assessed by examining the products and pathways of its degradation.

## 2. Results and discussion

### 2.1. Monoclinic and cubic phases of CsSnCl_3_

**Fig. 1:**
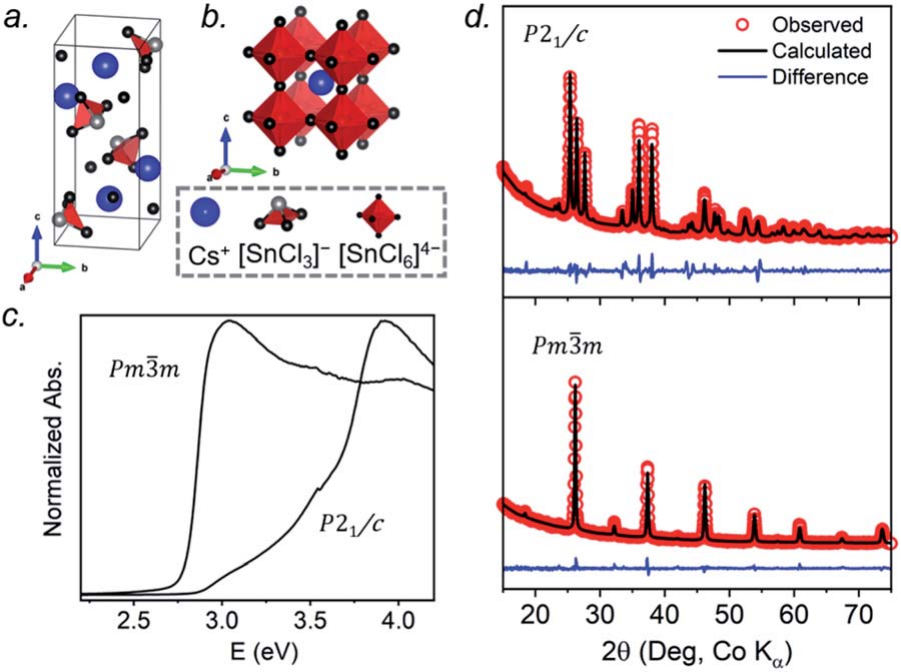
CsSnCl_3_: (a) monoclinic structure at room temperature, (b) cubic perovskite structure above 379 K, (c) UV-visible absorption spectra collected at room temperature, and (d) Rietveld refinements of powder XRD patterns.

At room temperature, CsSnCl_3_ adopts a monoclinic structure (space group *P*2_1_/*c*) containing isolated [SnCl_3_]^−^ ions in trigonal pyramidal geometry with Sn–Cl bonds of 2.50–2.55 Å (Fig. 1a). If three much more distant Cl atoms at 3.21–3.77 Å are included, the coordination geometry around the Sn atoms (coordination number (CN) with 3 shorter and 3 longer bonds, CN3 + 3) could also be described as distorted octahedral.^21^ Above 379 K, CsSnCl_3_ transforms to the cubic perovskite structure (space group *Pm*3̄*m*) containing a network of corner-sharing ideal [SnCl_6_]^4−^ octahedra with Sn–Cl bonds of 2.78 Å (Fig. 1b).^21,50^ However, as described later, the cubic form can be obtained at room temperature as a metastable phase. From le Bail fitting of the powder XRD patterns (Fig. 1d), the cell parameters were refined to be *a* = 5.7286(6) Å, *b* = 7.6936(11) Å, *c* = 16.9175(15) Å, and *β* = 106.505(4)° for the monoclinic phase and *a* = 5.5894(5) Å for the room temperature cubic phase, in good agreement with previously reported values.^50,51^ The room temperature UV-visible absorption spectra show that the monoclinic phase has a larger optical band gap (3.33 eV) than the cubic phase (2.83 eV) (Fig. 1c), consistent with the less delocalized electronic structure associated with the discrete [SnCl_3_]^−^ units in the former.

The lack of a simple relationship between the monoclinic and cubic structures of CsSnCl_3_ implies a reconstructive phase transition involving considerable mobility of atoms, because the process takes place at a relatively low temperature. In preparation for a detailed examination of the dynamics of this process, the local environments around the Cs and Sn atoms within the monoclinic and cubic phases were first probed by solid-state NMR spectroscopy to establish a frame of reference.

**Fig. 2:**
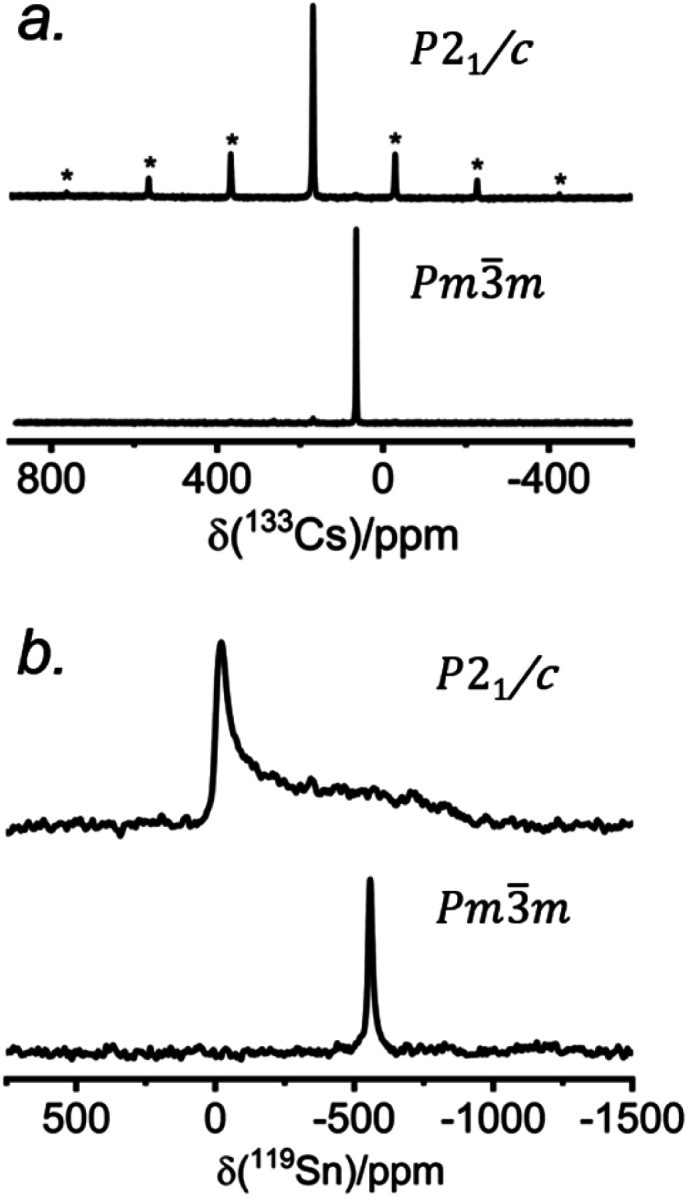
(a) ^133^Cs and (b) ^119^Sn NMR spectra collected at room temperature for monoclinic and cubic CsSnCl_3_. The ^133^Cs NMR spectra were acquired at 11.75 T with a magic-angle spinning frequency of 13 kHz. The ^119^Sn NMR spectra were acquired at 7.05 T for monoclinic and 11.75 T for cubic CsSnCl_3_ under non-spinning sample conditions at 293 K. In (a), the asterisks (*) indicate spinning side bands.

The ^133^Cs NMR characteristics depend strongly on local electronic and chemical environments. The position and widths of the ^133^Cs resonances, as measured by their isotropic chemical shift (*δ*_iso_) and full-width-at-half-maximum (fwhm) values, are quite different within the monoclinic (*δ*_iso_ = 168.5(1) ppm, fwhm = 320 Hz) and cubic structures (*δ*_iso_ = 64.7(1) ppm, fwhm = 138 Hz) (Fig. 2a). The ^133^Cs spin-lattice relaxation time decreases significantly from the monoclinic (*T*_1_ = 252 s) to the cubic structure (*T*_1_ = 15 s), which may be related to greater mobility of Cl atoms in the latter.^21^ The local environment of Cs atoms surrounded by Cl atoms is less symmetrical within the monoclinic structure (CN10, bicapped square prismatic), giving rise to a manifold of spinning sidebands due to a small quadrupole coupling constant (*C*_Q_) of 190 kHz, whereas it is highly symmetrical within the cubic structure (CN12, cuboctahedral) for which *C*_Q_ is ∼0 kHz. At a magic-angle spinning (MAS) frequency of 13 kHz, a single low-intensity spinning side band is observed in cubic CsSnCl_3_ (Fig. S1†), which is most likely due to intrinsic defects (*e.g.*, Cl vacancies).^52^ The distinctly different ^133^Cs NMR spectra for these two forms of CsSnCl_3_ can then be exploited to quantitatively determine their relative amounts in more complex samples obtained by various synthetic procedures.

Similarly, the ^119^Sn NMR features serve as helpful diagnostics of these forms of CsSnCl_3_ (Fig. 2b). The lower symmetry monoclinic form exhibits a broad pattern characteristic of chemical shift anisotropy (*δ*_iso_ = −295(2) ppm, span *Ω* = 870(5) ppm, skew *k* = 0.95(2)), whereas the higher symmetry cubic form exhibits a symmetric resonance (*δ*_iso_ = −560(1) ppm, fwhm = 1.8 kHz) with no evidence for chemical shift anisotropy.

### 2.2. Trapping cubic CsSnCl_3_ at room temperature

According to recent reports, cubic CsSnCl_3_ can be obtained as a metastable phase at room temperature by ball milling,^53^ or by “briefly heating” the monoclinic form to 380 K.^44^ However, questions still remain about how long this cubic phase persists under ambient conditions, and to our knowledge, there has been no systematic study on its stability upon exposure to air.

**Fig. 3:**
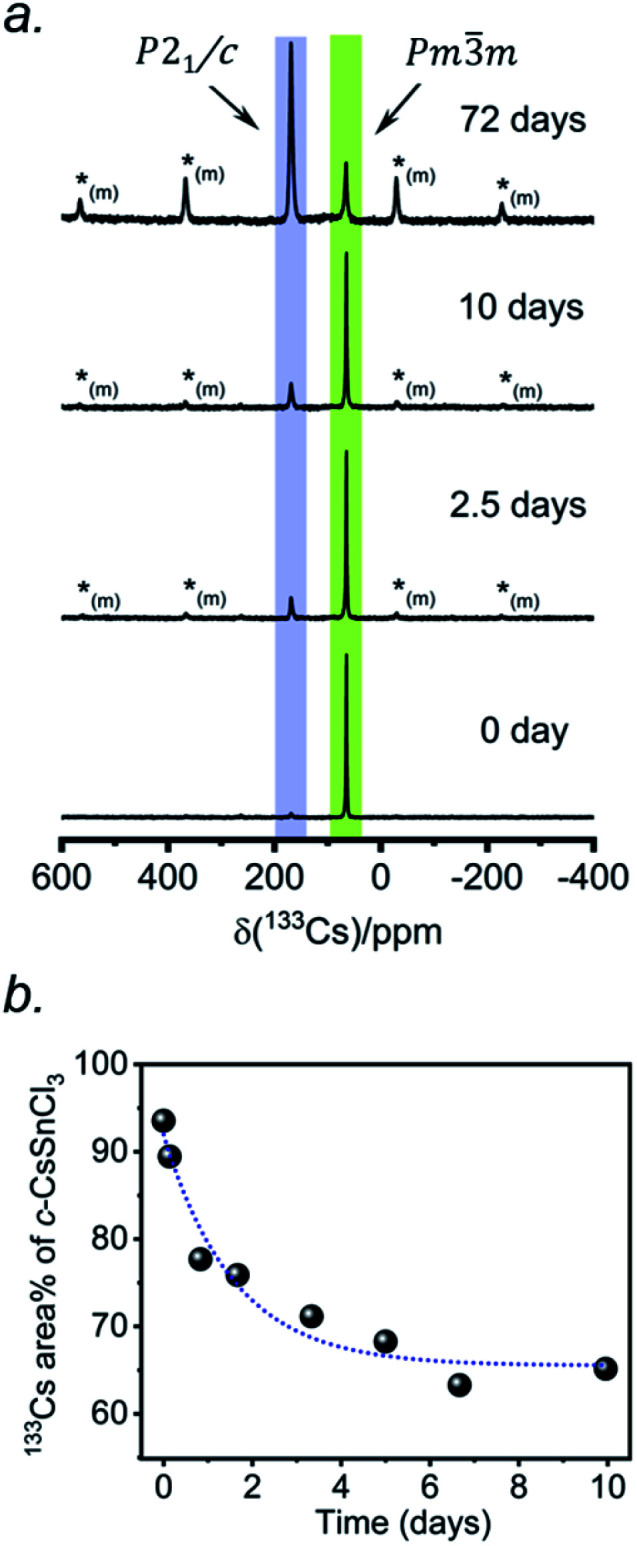
(a) Time-dependent ^133^Cs NMR spectra (*B*_0_ = 11.75 T, *ω*_r_/2π = 13 kHz MAS; a short-tip angle and an optimized 300 s recycle delay were used for quantification) of a sample of CsSnCl_3_ prepared by quenching in ice-water. The spectra are normalized to the highest intensity. The asterisks (*_(m)_) indicate positions of spinning side bands for the monoclinic phase. (b) Plot of ^133^Cs NMR peak area (±2%) as a function of time for the cubic phase.

Two separate samples of CsSnCl_3_ were prepared by reaction of CsCl and SnCl_2_, which were loaded into sealed and evacuated fused-silica tubes, and heated at 673(10) K for 15 h in a box furnace (see Experimental section in ESI†). For the first sample, the tubes were cooled slowly at 5 K min^−1^ to room temperature. This sample contained a mixture of cubic and monoclinic phases with the latter >50%, as revealed by both powder XRD and ^133^Cs NMR spectroscopy performed within 24 hours of synthesis (Fig. S2†). After 6 days at room temperature, the sample transformed to mostly the monoclinic phase, with <10% of the cubic phase remaining. For the second sample, the tubes were cooled at 5 K min^−1^ to 573 K, followed by quenching in an ice-water bath. Although the powder XRD data suggest a phase-pure sample consisting of only the cubic phase (lower part of Fig. 1d), the ^133^Cs NMR spectrum shows a small amount (6%) of the monoclinic phase (Fig. 3a). Time-dependent ^133^Cs NMR spectra were collected on the sample, kept at room temperature, at various intervals up to 72 days. The proportion of the cubic phase decreases monotonically in an exponential manner, with 65% remaining after 10 days and 14% after 72 days (Fig. 3b and S2c†). This process is much slower than a similar one that we have recently reported for the transformation of metastable γ-CsPbI_3_ perovskite to δ-CsPbI_3_ non-perovskite at room temperature, which is complete within 2 hours, as monitored by ^133^Cs NMR spectroscopy.^41^

### 2.3. Cubic CsSn(Cl_1−*x*_Br_*x*_)_3_ solid solution: synthesis, long-range structure and optical properties

The multiple phase transitions encountered for both CsSnCl_3_ and CsSnBr_3_ make it challenging to prepare a complete mixed-halide solid solution CsSn(Cl_1−*x*_Br_*x*_)_3_ having the cubic perovskite structure over the entire range for *x* at room temperature. Moreover, the parent end-members only adopt the stable cubic structure above room temperature (>379 K for CsSnCl_3_ and >292 K for CsSnBr_3_).^21,22^ Samples of CsSn(Cl_1−*x*_Br_*x*_)_3_ prepared by solution methods were reported to form monoclinic phases for Cl-rich compositions (*x* < 0.50) and cubic phases for Br-rich compositions (*x* > 0.50) at room temperature.^16^ The retention of cubic CsSnCl_3_ as a metastable phase at room temperature, presented above, suggests that it may yet be possible to prepare a complete solid solution with the cubic structure through similar high-temperature reactions of CsX and SnX_2_ (X = Cl, Br). As detailed in the experimental section, the critical step is rapid quenching (see ESI†).

**Fig. 4:**
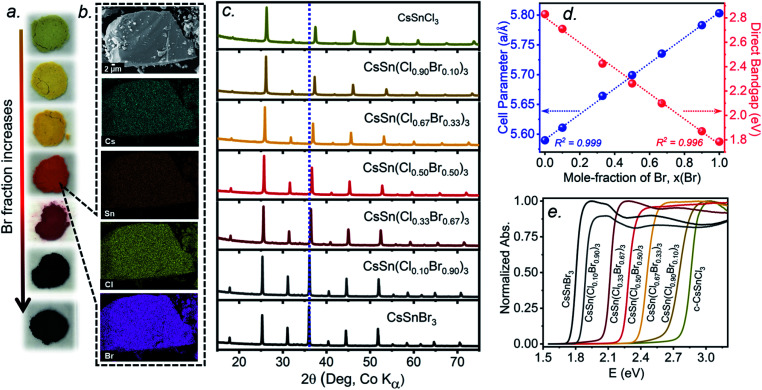
(a) Photographs of freshly synthesized CsSn(Cl_1−*x*_Br_*x*_)_3_ (top to bottom, *x* = 0, 0.10, 0.33, 0.50, 0.67, 0.90, 1), (b) scanning electron micrograph and elemental maps for CsSn(Cl_0.50_Br_0.50_)_3_, (c) powder XRD patterns, (d) plots of unit cell parameters and band gaps, and (e) UV-visible absorption spectra.

Freshly synthesized samples of CsSn(Cl_1−*x*_Br_*x*_)_3_ show colours starting from faint yellow (CsSnCl_3_) and gradually evolving with increasing Br content to orange, red, and finally black (CsSnBr_3_), consistent with a decrease in the band gap (Fig. 4a). The samples are polycrystalline with micron-sized particles having uniform distribution of all elements (Cs, Sn, Cl, Br) down to this scale, as seen in elemental mapping images collected on a field-emission scanning electron microscope (Fig. 4b and S3†). The chemical compositions, as determined by energy-dispersive X-ray analysis, agree well with the nominal compositions CsSn(Cl_1−*x*_Br_*x*_)_3_ loaded in the reactions (Table S1†).

Powder XRD patterns collected at room temperature confirm the cubic perovskite structure (space group *Pm*3̄*m*) for all members of the solid solution (Fig. 4c and S4†). The refined unit cell parameter for CsSn(Cl_1−*x*_Br_*x*_)_3_ increases linearly from 5.5894(5) Å for CsSnCl_3_ to 5.8031(3) Å for CsSnBr_3_, in accordance with Vegard's law, with no deviations being shown (Fig. 4d and Table S2†). Strict adherence to Vegard's law behaviour has also been observed in lead-containing mixed-halide perovskites.^35,36^

UV-visible absorption spectra, which were converted from diffuse reflectance spectra using the Kubelka–Munk function, show gradual shifts in the absorption edge to lower energy with increasing Br content in CsSn(Cl_1−*x*_Br_*x*_)_3_ (Fig. 4e). With the assumption of a direct band gap, the linear regions in the Tauc plots of (*αhν*)^2^*vs. E* were extrapolated to extract optical band gap values (Fig. S5†). The band gap in CsSn(Cl_1−*x*_Br_*x*_)_3_ decreases linearly with increasing Br content from 2.83 eV for CsSnCl_3_ to 1.79 eV for CsSnBr_3_ (Fig. 4d), similar to the behaviour seen in lead-containing mixed-halide perovskites.^35,36,41^

### 2.4. Cubic CsSn(Cl_1−x_Br_x_)_3_ solid solution: local structure using ^133^Cs and ^119^Sn NMR spectroscopy

**Fig. 5:**
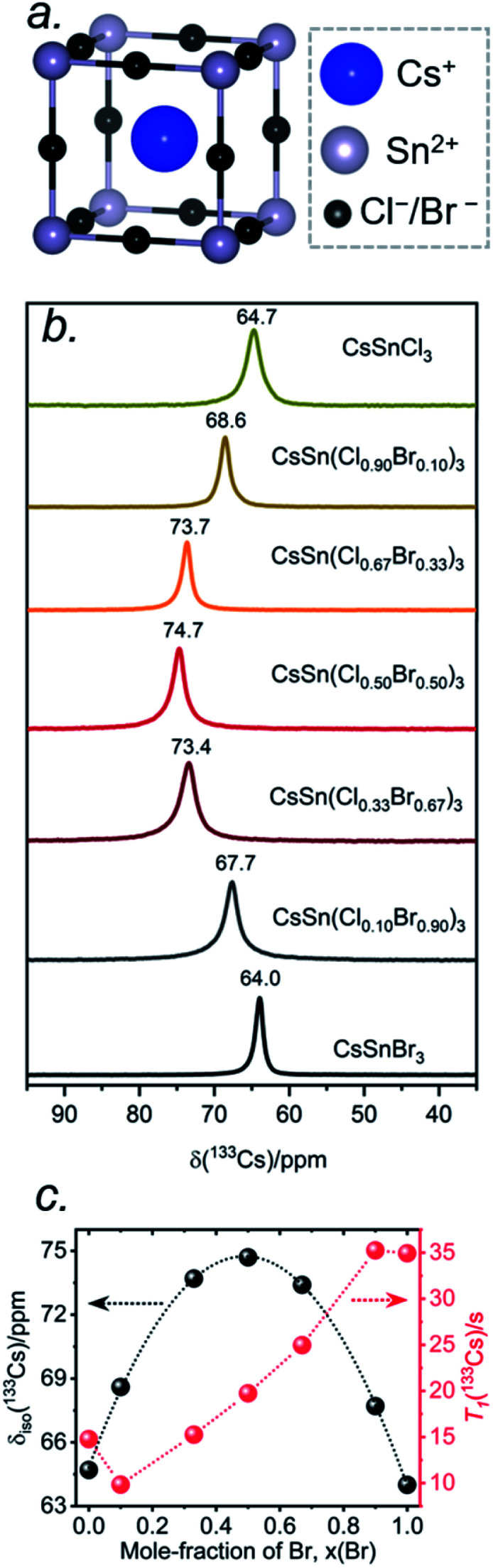
(a) Local cuboctahedral environment of 12 halogen atoms around the Cs atom. (b) Room temperature ^133^Cs NMR spectra for CsSn(Cl_1−*x*_Br_*x*_)_3_, acquired at 11.75 T with a magic-angle spinning frequency of 13 kHz. (c) Plots of *δ*_iso_(^133^Cs) and ^133^Cs *T*_1_ values as a function of Br content. The *δ*_iso_ plot was fit to the equation *δ*_iso_(^133^Cs)/ppm = 64.8 + 40.6*x* − 41.5*x*^2^ (*R*^2^ = 0.999).

The local environments of Cl and Br atoms around the Cs and Sn atoms in CsSn(Cl_1−*x*_Br_*x*_)_3_ were probed by NMR spectroscopy. The Cs atoms reside in a cuboctahedral site surrounded by 12 halogen atoms (Fig. 5a). For the end-members, the ^133^Cs chemical shifts are similar but distinguishable: *δ*_iso_ = 64.7 ppm for CsSnCl_3_ and *δ*_iso_ = 64.0 ppm for CsSnBr_3_.^44^ Interestingly, the ^133^Cs resonances are quite sharp for all members of CsSn(Cl_1−*x*_Br_*x*_)_3_, with fwhm ranging from 70 to 150 Hz (or 1.1 to 2.3 ppm) (Fig. 5b). This result is in contrast to the lead-containing analogues CsPb(Cl_0.50_Br_0.50_)_3_, where much broader ^133^Cs resonances (fwhm of 2 kHz) were observed and attributed to inhomogeneities caused by the numerous possible arrangements of Cl and Br atoms.^41^ The sharper ^133^Cs NMR spectra for CsSn(Cl_1−*x*_Br_*x*_)_3_ imply that rapid halogen dynamics must be taking place even at room temperature, such that the effects of the 12 surrounding halogen atoms are averaged.^44^

Most surprisingly, the ^133^Cs chemical shift does not vary monotonically between the end-members; rather, it is displaced to higher frequency relative to the end-members and reaches a maximum of *δ*_iso_ = 74.7 ppm in CsSn(Cl_0.50_Br_0.50_)_3_. The relationship between the chemical shift *δ*_iso_ and the Br content *x* can be fitted to a parabolic curve, following the quadratic function *δ*_iso_(^133^Cs)/ppm = 64.8 + 40.6*x* − 41.5*x*^2^ (Fig. 5c). A comparable nonlinear change in ^89^Y and ^119^Sn NMR chemical shifts has been observed in pyrochlores.^54,55^ A similar nonlinear change in ^133^Cs NMR chemical shifts was recently reported for mixed-halide double perovskites Cs_2_AgBi(Cl_1−*x*_Br_*x*_)_6_ due to a non-additive effect of nearest and next-nearest neighbours on the chemical shift for ^133^Cs nuclei and the associated extended halide environment.^56^

A mixture of Cl and Br atoms in the cuboctahedral environment around the Cs atoms would be expected to enhance quadrupolar coupling interactions because of the lowering of symmetry and correspondingly to increase the manifold of ^133^Cs spinning side bands, as was previously observed in the lead-containing analogues CsPb(Cl_0.50_Br_0.50_)_3_.^41^ However, at a MAS frequency of 13 kHz, only a single spinning side band of low intensity was observed for all members of CsSn(Cl_1−*x*_Br_*x*_)_3_ (Fig. S1†), which could occur if the environment contains identical halogen atoms (not possible here) or if the halogen dynamics are sufficiently rapid (*i.e.*, much shorter than the correlation times) such that only average values are measured for the relevant interactions. The ^133^Cs spin-lattice relaxation times *T*_1_ vary nonlinearly between 10 to 35 s with increasing Br content (Fig. 5c and Table S3†). These short relaxation times, on the order of seconds, are consistent with the absence of significant covalent bonding interactions between Cs and halogen atoms, similar to previous observations for CsPb(Cl_0.50_Br_0.50_)_3_.^42^

**Fig. 6:**
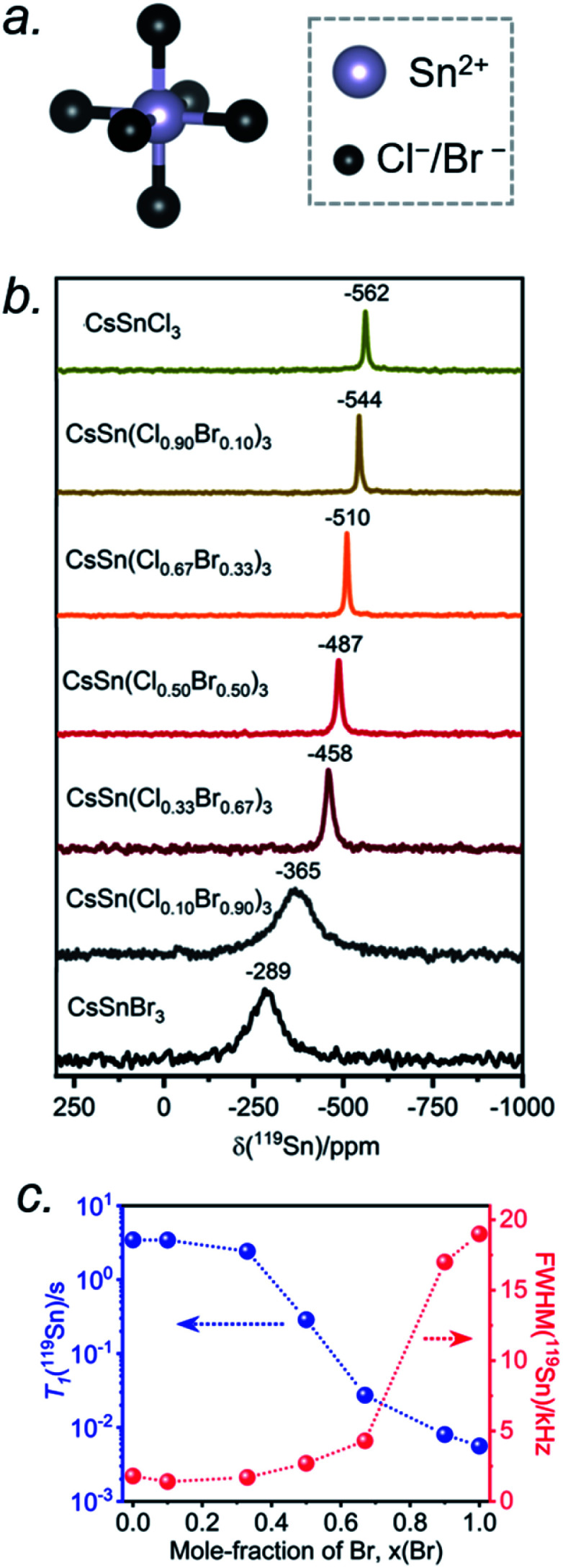
(a) Local octahedral environment of 6 halogen atoms around the Sn atom. (b) Room-temperature ^119^Sn NMR spectra for CsSn(Cl_1−*x*_Br_*x*_)_3_, acquired at 11.75 T with a magic-angle spinning frequency of 10 kHz. (c) Plots of ^119^Sn *T*_1_ and ^119^Sn linewidth values as a function of Br content.

The Sn atoms in CsSn(Cl_1−*x*_Br_*x*_)_3_ reside in an octahedral site surrounded by six halogen atoms (Fig. 6a). For the end-members, the ^119^Sn NMR spectra show symmetric-like lineshapes with clear differences in isotropic chemical shifts for CsSnCl_3_ (*δ*_iso_ = −562 ppm) and CsSnBr_3_ (*δ*_iso_ = −289 ppm) (Fig. 6b). There is no evidence of spinning side bands, consistent with a highly symmetric chemical environment. As the Br content increases, the ^119^Sn chemical shift gradually displaces to higher frequency (Fig. 6b and Table S4†) and the linewidth increases from CsSnCl_3_ (fwhm = 1.8 kHz) to CsSnBr_3_ (fwhm = 19.0 kHz) (Fig. 6c). For CsSnBr_3_, the ^119^Sn NMR linewidth and lineshape are unchanged whether the magnetic field (*B*_0_) is 7.05 or 11.75 T (Fig. S6†). Furthermore, the linewidth (fwhm = 21 kHz) only decreases slightly when magic angle spinning is applied (fwhm = 19 kHz) (Fig. S7†); the small decrease is attributed to a reduced heteronuclear dipolar coupling between ^119^Sn and ^79/81^Br. The observed linewidths may also be influenced by indirect spin–spin coupling between ^119^Sn and six surrounding quadrupolar nuclei (*I* = 3/2 for ^35/37^Cl and ^79/81^Br) as recently reported in CH_3_NH_3_PbCl_3_ and CsPbCl_3_.^24,28^ For the Br-rich samples, the ^119^Sn signal-to-noise ratio drastically deteriorates upon increasing the echo delays from 24 to 94 μs, due to fast *T*_2_ relaxation (Fig. S8†).^44^ These results are similar to previous observations on the lead-containing analogues.^24,28,31,35,36,41^

The occurrence of single symmetric-like ^119^Sn resonances and the absence of spinning side bands imply that the chemical environment around the Sn atoms is uniform. If a distribution of several types of SnCl_6−*n*_Br_*n*_ (*n* = 0–6) octahedra were present, they would give rise to multiple Sn resonances and possibly spinning side bands due to magnetic shielding anisotropy, but neither of these are observed. As before, the most probable explanation is that rapid halogen dynamics is taking place in CsSn(Cl_1−*x*_Br_*x*_)_3_ on the timescale of the NMR experiment, resulting in averaged chemical environments around the Sn atoms. This situation contrasts with the lead-containing analogues CsPb(Cl_1−*x*_Br_*x*_)_3_, where multiple chemical environments around Pb atoms are observed.^41^ The ^119^Sn NMR chemical shift can be further correlated with the band gap in CsSn(Cl_1−*x*_Br_*x*_)_3_ (Fig. S9†). The decrease in band gap is accompanied by a displacement of the ^119^Sn NMR chemical shift to higher frequency, suggesting a dominating paramagnetic shielding contribution.

The ^119^Sn spin-lattice relaxation times decrease dramatically from CsSnCl_3_ (*T*_1_ = 3.5 s) to CsSnBr_3_ (*T*_1_ = 0.006 s), a difference of three orders of magnitude which is comparable to recent observations on CH_3_NH_3_SnX_3_.^44^ The decrease is monotonic with increasing Br content (Fig. 6c and Table S4†). It has been shown previously that the *T*_1_ relaxation mechanism is dominated by the scalar coupling strength ^1^*J*(^119^Sn, X) (X = ^35/37^Cl or ^79/81^Br) for tin halides.^44,57,58^ The smaller *T*_1_ value for CsSnBr_3_ compared to CsSnCl_3_ is consistent with ^1^*J*(^119^Sn,^79/81^Br) being larger than ^1^*J*(^119^Sn,^35/37^Cl). In addition to *J*-coupling, chemical exchange processes due to the rapid halogen dynamics at room temperature may also play a significant role in the ^119^Sn *T*_1_ values for the Br-rich samples, as discussed later.

### 2.5. Local structure of CsSnBr_3_ prepared by solvent, high-temperature, and mechanochemical routes

There have been many reports of halide perovskites being prepared by nonconventional routes, either in the presence or absence of solvent. For example, solvent-free “mechanochemical synthesis” routes involving ball-milling have been proposed to be advantageous for large-scale production of perovskite photovoltaic materials.^59,60^ However, it is unclear whether samples prepared by these nonconventional routes are really identical to more traditional solution-based methods on a local atomic-level. To understand this, samples of CsSnBr_3_ were prepared by three routes (solvent synthesis, high-temperature reaction, and ball-milling) and analyzed by ^133^Cs and ^119^Sn NMR spectroscopy.

**Fig. 7:**
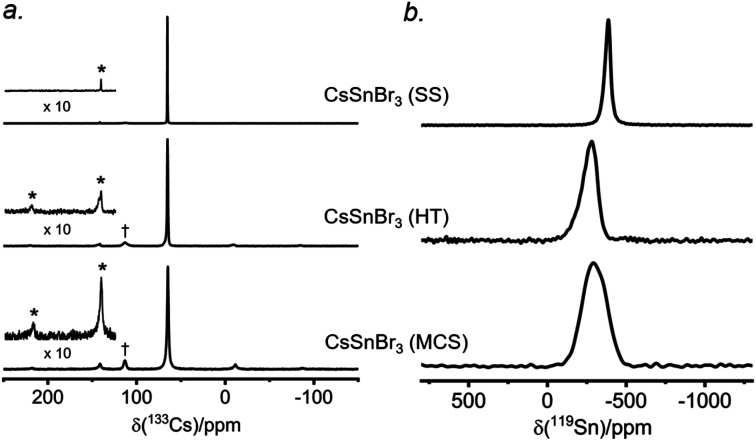
Room temperature (a) ^133^Cs NMR spectra with magic-angle spinning (5 kHz) and (b) ^119^Sn NMR spectra with no magic-angle spinning, acquired at 11.75 T, of CsSnBr_3_ samples prepared by solvent synthesis (SS), high-temperature (HT) reactions, and ball-milling (MCS). In (a), the asterisks (*) indicate spinning side bands and the daggers (†) mark peaks attributed to Cs_2_SnBr_6_.

The ^133^Cs NMR spectra show resonances appearing in the same position, being sharpest for the sample prepared by solvent-synthesis (fwhm = 15 Hz), and growing increasingly broader for those prepared by high-temperature reaction (fwhm = 70 Hz) and ball-milling (fwhm = 120 Hz) (Fig. 7a). For these spectra acquired under slow magic-angle spinning (5 kHz), the spinning side band manifold increases along the same order. These observations indicate that the solvent-synthesized sample has the most well-ordered structure containing fewer defects, whereas the ball-milled sample has the most locally disordered structure exhibiting a wide distribution of environments and more defects.^27,35^ The higher defect concentrations in our ball-milled sample are maintained because no further post-grinding annealing treatment was performed. It is worth noting that one may minimize or circumvent the formation of grinding induced defects by performing a subsequent heat treatment after ball milling.^61,62^

The ^119^Sn NMR spectra for these samples (Fig. 7b) show even greater differences in chemical shifts, which span a range of almost 100 ppm, and linewidths: the sharpest at −386 ppm (fwhm = 7.5 kHz) for the solvent-synthesized sample, an intermediate one at −284 ppm (fwhm = 21 kHz) for the sample prepared at high temperature, and the broadest at −295 ppm (fwhm = 34 kHz) for the ball-milled sample. The ^119^Sn spin-lattice relaxation times depend greatly on the synthetic route: *T*_1_ = 25 ms for the solvent-synthesized sample, 7 ms for the high-temperature sample, and 3 ms for the ball-milled sample (Table S5†). A faster relaxation process implies greater local surface disorder/defects in the ball-milled sample, consistent with the conclusions from the ^133^Cs NMR spectra and the ^119^Sn NMR linewidths. This greater disorder is also reflected in broader peaks in the powder XRD patterns for the ball-milled sample compared to the other samples (Fig. S10†); all samples exhibit micron sized crystallites. However, the optical band gaps extracted from the UV-visible absorption spectra appear to be virtually identical (within experimental error), with values ranging from 1.77 eV for the solution-synthesized sample to 1.81 eV for the ball-milled sample. We note this disorder may also be related to the UV-vis absorption profile being different for the ball-milled sample (Fig. S11†).

### 2.6. Spontaneous halogen mobility in CsSnBr_3_

**Fig. 8:**
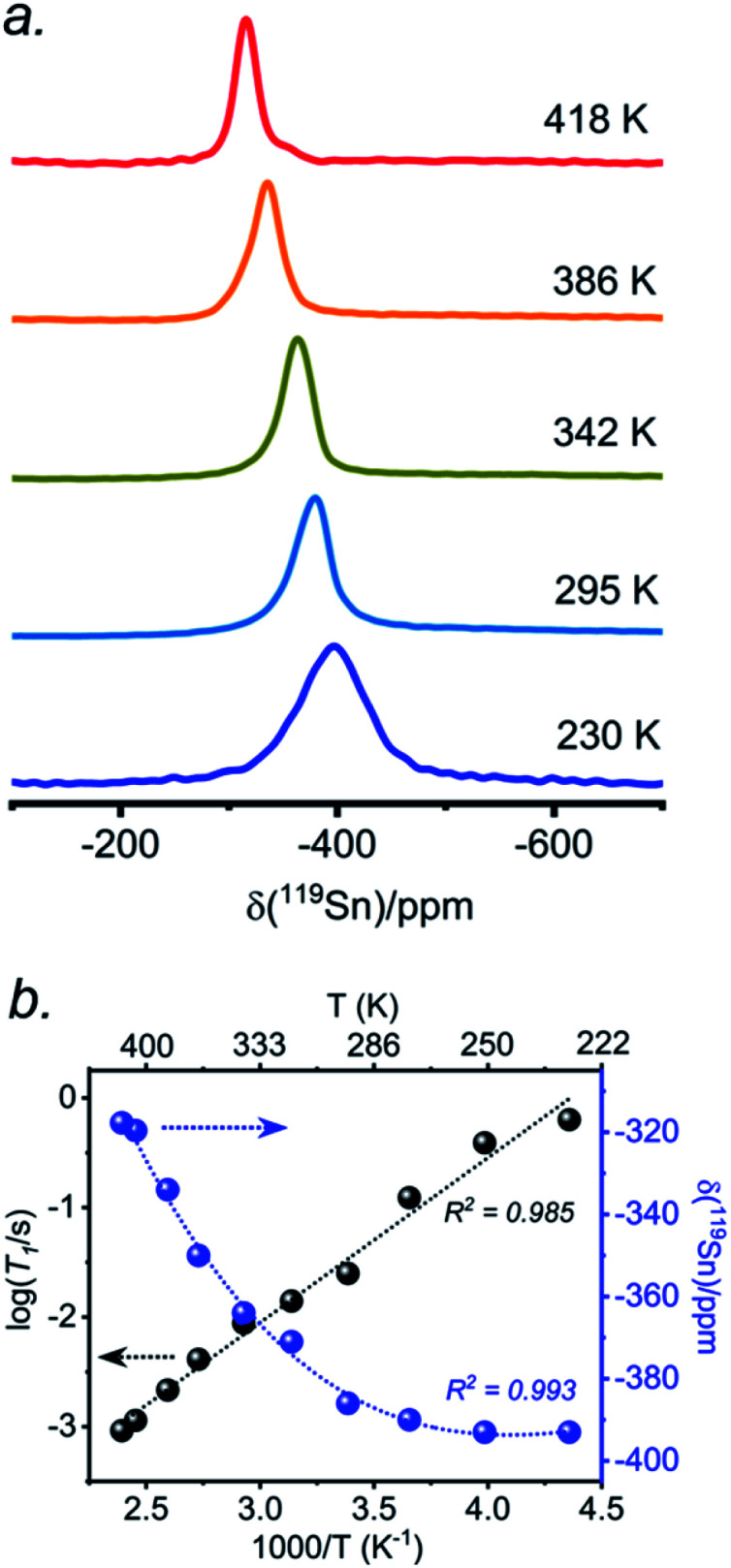
(a) Variable temperature ^119^Sn NMR non-spinning spectra acquired at 11.75 T and (b) plots of ^119^Sn *T*_1_ and NMR chemical shift *vs.* inverse of absolute temperature for solvent-synthesized CsSnBr_3_.

The presence of point defects or vacancies enables ionic migration within perovskites and plays an important role in photovoltaic efficiency and material stability, while also being responsible for photocurrent hysteresis characteristics.^63–65^ Metal halide perovskites ABX_3_ can exhibit a vacancy transport hopping mechanism through A-, B- or X-site migration,^66^ but X-site (halogen) diffusion is most probable as the halogen vacancy formation energy is comparable to the halogen ionic diffusion activation energy barrier (*ca.* 0.30 eV) in organic–inorganic hybrid perovskites.^52,67,68^ The analysis of the ^133^Cs and ^119^Sn NMR spectra above suggests that halogen atoms may be highly mobile in CsSn(Cl_1−*x*_Br_*x*_)_3_ even at room temperature. To deconvolute the halogen dynamics, variable-temperature ^119^Sn NMR spectroscopy was performed on the parent CsSnBr_3_ compound, prepared by solution synthesis. As the temperature increases from 230 to 418 K, the ^119^Sn resonances shift monotonically to higher frequency by about 75 ppm (Fig. 8 and Table S6†), following the cubic dependence 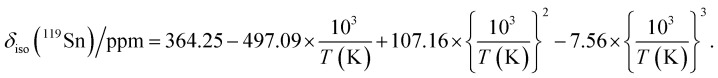 The ^119^Sn NMR linewidth narrows from 14.7 to 4.5 kHz, the effective spin–spin relaxation time 
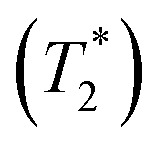
 increases from 68 to 222 μs (Fig. S12 and Table S6†), and most notably, the spin-lattice relaxation time (*T*_1_) decreases drastically from 0.635 to 0.0009 s, a difference of three orders of magnitude (Table S6†). In accordance with an Arrhenius relationship, a plot of log_10_[*T*_1_(^119^Sn)/s] *vs.* [1000/*T* (K)] yields a linear dependence (Fig. 8b) from which an activation energy of 28.9 ± 1.2 kJ mol^−1^ (0.30 ± 0.01 eV) for the halogen dynamics was extracted (see ESI Note 1†). Although this result is in contrast to the metallic behaviour previously suggested from electrical conductivity measurements,^67^ it is in excellent agreement with those for other Sn- and Pb-based perovskites and metal halide solid ionic conductors including CH_3_NH_3_SnBr_3_ (0.30–0.37 eV),^44,67,68^ CH_3_NH_3_PbI_3_ (0.29 eV),^69^ CsPbCl_3_ (0.29 eV),^70^ CsPbBr_3_ (0.25 eV),^70^ CsSnCl_3_ (0.2 eV, theoretical),^71^ α-SnI_2_ (0.29 eV),^72^ PbCl_2_ (0.20–0.30 eV),^70,73^ and PbBr_2_ (0.23–0.25 eV).^70,74^ These observations support the conclusion that CsSnBr_3_ behaves like an ionic conductor and Br^−^ mobility is responsible for the fast relaxation process of ^119^Sn nuclei in CsSnBr_3_, as observed recently in the organic–inorganic hybrid CH_3_NH_3_SnBr_3_ perovskite.^44^

The above NMR results suggest that the replacement of Pb with Sn in perovskite appears to enable faster halogen mobility, which causes an averaging of the relevant interactions through halide dynamics. The faster halogen dynamics alter the electrical properties for tin halide perovskites. For example, cubic CsSnCl_3_ was shown to have high ionic conductivity >10^−4^ S cm^−1^ at 313 K, fueling its potential as a solid electrolyte candidate for chloride ion batteries.^71^ We further note that a higher concentration of B site vacancies (*ca.* 2.9%)^71^ was reported in CsSnCl_3_ than that for CH_3_NH_3_PbI_3_ (*ca.* 0.4%).^75^ The higher B site vacancies in tin halide perovskites is most likely associated with Sn(+2) oxidation (Sn(+2) → Sn(+4) *vs.* Pb(+2)), which may cause an increase in the rate of halogen dynamics.

### 2.7. Air stability and degradation pathways for CsSnBr_3_

**Fig. 9:**
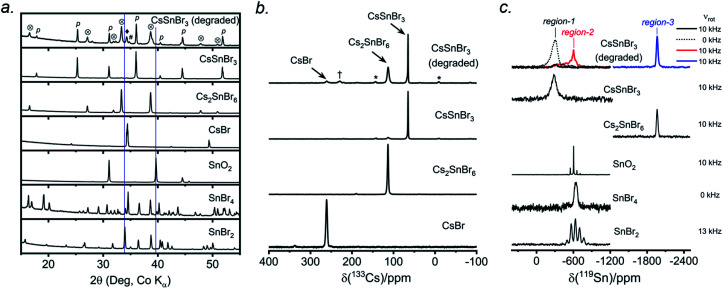
Degraded CsSnBr_3_ sample that was exposed to ambient laboratory conditions over 300 days. (a) Powder XRD pattern compared to reference standards shows presence of CsSnBr_3_ (p), Cs_2_SnBr_6_ (⊗), CsBr (♦), and SnBr_4_ (#). The blue lines indicate the absence of peaks due to SnO_2_ and SnBr_2_. (b) ^133^Cs NMR spectrum, acquired at 11.75 T with a magic-angle spinning frequency of 5 kHz, shows the presence of CsSnBr_3_, Cs_2_SnBr_6_, and CsBr. The asterisks (*) indicate spinning side bands and the dagger (†) indicates an unidentified Cs-containing degradation product. (c) ^119^Sn NMR spectrum, acquired at 11.75 T with magic-angle spinning frequencies of 0 to 13 kHz.

CsSnBr_3_ shows higher thermal and moisture stability than the organic–inorganic hybrid CH_3_NH_3_SnBr_3_. Nevertheless, CsSnBr_3_ slowly degrades over time under ambient conditions, becoming partially oxidized to Cs_2_SnBr_6_, and the UV-visible absorption spectra change drastically (Fig. S13†). Powder XRD and solid-state ^133^Cs and ^119^Sn NMR spectroscopy were performed to elucidate the decomposition pathways and the nature of the degraded products. A sample of CsSnBr_3_ was freshly synthesized by high-temperature reactions and stored in a parafilm-sealed glass vial under ambient laboratory conditions (298 K and 50–70% humidity) over 300 days. The powder XRD pattern of the degraded sample was compared to those for various reference standards (pristine CsSnBr_3_, Cs_2_SnBr_6_, CsBr, SnO_2_, SnBr_4_, and SnBr_2_) (Fig. 9a). In addition to CsSnBr_3_, which still constituted about 40% of the sample, significant amounts of Cs_2_SnBr_6_ and CsBr, and smaller amounts of SnBr_4_ were formed, but no SnO_2_ or SnBr_2_ were present. The powder XRD pattern also shows an increased background, suggestive of amorphous contributions.

Complementary information about crystalline and amorphous components can be obtained from the solid-state NMR spectra. For quantitative analysis, the NMR parameters were optimized to achieve maximum sensitivity (Tables S7 and S8†). The ^133^Cs NMR spectrum of the degraded sample was compared to those for three reference standards (pristine CsSnBr_3_, Cs_2_SnBr_6_, and CsBr) (Fig. 9b). Based on assignment of the ^133^Cs NMR resonance areas, the sample consisted of 40% CsSnBr_3_ (65 ppm), 45% Cs_2_SnBr_6_ (113 ppm), 8% CsBr (260 ppm) and 7% of an unidentified Cs-containing phase (230 ppm). The ^133^Cs resonances are slightly broader for Cs_2_SnBr_6_ (fwhm of 320 Hz, compared to 170 Hz for the solvent-synthesized standard) and for CsBr (fwhm of 370 Hz, compared to 270 Hz for the standard). This broadening implies that Cs_2_SnBr_6_ and CsBr formed during the slow degradation process have smaller crystallite sizes than in the reference standards.^44^ The powder XRD pattern also shows broadening of diffraction peaks for these phases (Fig. 9a).

In the ^119^Sn NMR spectrum of the degraded sample, the resonances span about 8800 ppm (1.6 MHz at *B*_0_ = 11.75 T, Fig. 9c and S14†), with spin-lattice relaxation times extending over four orders of magnitude (Table S8†). Hence, the ^119^Sn NMR acquisition parameters were optimized within different spectral regions (Table S8†). Region 1 shows that a significant amount of CsSnBr_3_ is present. The ^119^Sn resonance intensity for CsSnBr_3_ is highly sensitive to the magic-angle spinning frequency; because these spectra were acquired with a spin echo, for which the delay depends on the spinning frequency, the signal is weakened due to the fast *T*_2_ relaxation process when slower MAS frequencies are used. Region 2 shows a ^119^Sn resonance at −600 ppm, which corresponds to SnO_2_, and a broad underlying component, which most likely corresponds to SnBr_4_, indicating that oxidation from Sn(+2) to Sn(+4) has taken place. The ^119^Sn signal for SnO_2_ in the degradation product is much broader (fwhm = 3.7 kHz) than in the bulk SnO_2_ standard (fwhm = 0.2 kHz). Although crystalline SnO_2_ was not detected in the powder XRD pattern, the broadness of the ^119^Sn NMR signal suggests that it may either be amorphous or in the form of nanodomains (<10 nm)^76^ embedded within other phases in the sample. Region 3 confirms the presence of Cs_2_SnBr_6_, as seen by the ^119^Sn resonance at −1964 ppm. Region 4 reveals a weak ^119^Sn signal that is assigned to metallic β-Sn and is Knight-shifted^77^ to higher frequency; however, this signal was detected only after two million scans, implying only a trace amount of β-Sn that likely originated as an impurity in the starting material SnBr_2_ used in the synthesis, as shown in Fig. S14.† Based on the XRD and NMR results, two simultaneous pathways are proposed for the room-temperature oxidation of CsSnBr_3_:(1) 2 CsSnBr_3_ + O_2_ (air) → Cs_2_SnBr_6_ (crystalline) + SnO_2_ (amorphous or nano-sized)(2) 2 CsSnBr_3_ + O_2_ (air) → SnBr_4_ (crystalline) + 2 CsBr (crystalline) + SnO_2_ (amorphous or nano-sized).

## 3. Conclusions

The complete solid solution CsSn(Cl_1−*x*_Br_*x*_)_3_ (0 ≤ *x* ≤ 1) exhibiting the cubic perovskite structure for all members, was prepared by rapid quenching of samples reacted at high temperature. In particular, this method is able to trap the end-member CsSnCl_3_ as a metastable cubic phase (instead of the monoclinic phase) at room temperature. The long-range structure was elucidated by powder XRD and the local structure and dynamics by ^133^Cs and ^119^Sn solid-state NMR spectroscopy. In contrast to the lead-containing analogues CsPbX_3_, the halogen atoms in CsSn(Cl_1−*x*_Br_*x*_)_3_ are much more mobile. The rapid halogen dynamics in CsSnBr_3_ were analyzed by measuring the ^119^Sn spin-lattice relaxation times at variable temperatures, giving a low activation energy of 28.9 ± 1.2 kJ mol^−1^ for the diffusion of Br atoms. Although CsSnBr_3_ can be prepared by various synthetic routes that apparently have similar long-range structures, as ascertained by powder XRD and optical properties, the local structure and degree of crystallinity differ, as revealed by NMR spectroscopy; in particular, samples prepared by mechanochemical synthesis tend to show greater local polyhedral disorder and vacancies, resulting in a reduction of the spin-lattice relaxation of ^119^Sn. In a study of CsSnBr_3_ exposed to air, insight into the degradation pathways was sought by NMR spectroscopy, which revealed the formation of amorphous or nano-sized SnO_2_, not detected by conventional diffraction methods. The results suggest that although the photophysical properties of Sn-containing perovskites are attractive, and their band gaps are tunable through halide substitution and not highly affected by synthetic methods despite differences in local structure, challenges remain in ensuring their ambient long-term stability.

## Author's contribution

All authors have given approval to the final version of the manuscript.

## Conflicts of interest

There are no conflicts to declare.

## Supplementary Material

SC-012-D0SC05614F-s001
