# Supplementary material for: Influence of hidden halogen mobility on local structure of CsSn(Cl1−xBrx)3 mixed-halide perovskites by solid-state NMR
Source: Chem Sci. 2020 Dec 30;12(9):3253–63. doi: 10.1039/d0sc05614f (PMC8179406; doi:10.1039/d0sc05614f)
Supplement: SC-012-D0SC05614F-s001 [file SC-012-D0SC05614F-s001.pdf]

Electronic Supplementary Material (ESI) for Chemical Science.
This journal is © The Royal Society of Chemistry 2020

Electronic Supplementary Information (ESI)

Influence of Hidden Halogen Mobility on Local Structure of $CsSn(Cl_{1-x}Br_x)_3$ Mixed-Halide Perovskites by Solid-State NMR

Abhoy Karmakar, Amit Bhattacharya, Diganta Sarkar, Guy M. Bernard, Arthur Mar and Vladimir K. Michaelis*

Department of Chemistry, University of Alberta, Edmonton, Alberta T6G 2G2, Canada

**Corresponding Author: vladimir.michaelis@ualberta.ca*

Table of Contents	Page(s)
Experimental	S3-S5
**Table S1.** Elemental analysis of $CsSn(Cl_{1-x}Br_x)_3$ materials measured by EDS.	S5
**Table S2.** Unit cell constants (a) and direct bandgap values for $CsSn(Cl_{1-x}Br_x)_3$ materials.	S5
**Table S3.** Room temperature solid-state ^{133}Cs NMR experimental parameters, chemical shifts, fwhm and spin-lattice relaxation (T_1) values for $CsSn(Cl_{1-x}Br_x)_3$ materials.	S6
**Table S4.** Room temperature solid-state ^{119}Sn NMR experimental parameters, chemical shifts, fwhm and spin-lattice relaxation (T_1) values for $CsSn(Cl_{1-x}Br_x)_3$ materials.	S6
**Table S5.** Room temperature solid-state ^{119}Sn NMR experimental parameters (B_0 = 11.75 T), chemical shifts, fwhm and spin-lattice relaxation (T_1) for $CsSnBr_3$ materials prepared by the solvent synthesis (SS), high temperature (HT) and mechanochemical synthesis (MCS) methods.	S7
**Table S6.** Variable temperature ^{119}Sn NMR parameters for $CsSnBr_3$ (SS) at 11.75 T acquired under non-spinning sample conditions.	S7
Supplementary note 1	S7
**Table S7.** Solid-state ^{133}Cs NMR acquisition parameters and experimental conditions used for the $CsSnBr_3$ areal degradation study.	S8
**Table S8.** Solid-state ^{119}Sn NMR acquisition parameters and experimental conditions used for the $CsSnBr_3$ areal degradation study.	S8
**Figure S1.** Room temperature solid-state ^{133}Cs NMR spectra of $CsSn(Cl_{1-x}Br_x)_3$ materials at 11.75 T with a magic-angle spinning frequency of 13 kHz.	S9
**Figure S2.** Room temperature PXRD (a) and ^{133}Cs NMR spectra (b) of $CsSnCl_3$ materials prepared by the high-temperature sealed-tube method followed by slow-cooling (5 K/min). PXRD patterns were collected within 24 h of synthesis and ^{133}Cs NMR were acquired at 11.75 T after six days of synthesis. Plot of ^{133}Cs NMR peak area for the cubic $CsSnCl_3$ phase as a function of time (up to 72 days) (c).	S9
**Figure S3.** FESEM image and the corresponding EDS elemental mapping for Cs, Sn, Cl and Br for the $CsSn(Cl_{1-x}Br_x)_3$ materials.	S10
**Figure S4.** Room temperature experimental and fitted PXRD diagram for the $CsSn(Cl_{1-x}Br_x)_3$ series. All the diffraction data are fitted to a cubic (Pm-3m) space group symmetry.	S11
**Figure S5.** Tauc plots showing direct bandgaps of the cubic phases of $CsSn(Cl_{1-x}Br_x)_3$ materials.	S12
**Figure S6.** Solid-state ^{119}Sn NMR spectra of $CsSnBr_3$ at 7.05 and 11.75 T under non-spinning sample conditions.	S13
**Figure S7.** Solid-state ^{119}Sn NMR spectra of c-$CsSnCl_3$ (a), $CsSn(Cl_{0.50}Br_{0.50})_3$ (b) and $CsSnBr_3$ (c) at 11.75 T acquired with spinning frequencies of 0 and 10 kHz.	S13
**Figure S8.** Solid-state ^{119}Sn NMR spectra of $CsSn(Cl_{0.10}Br_{0.90})_3$ at 11.75 T acquired with spinning frequencies between 0 to 13 kHz with the Hahn-echo pulse sequence and with various echo-delays.	S14
**Figure S9.** Solid-state ^{119}Sn NMR chemical shifts vs the inverse of direct bandgap values for the $CsSn(Cl_{1-x}Br_x)_3$ series.	S14
**Figure S10.** Room temperature PXRD patterns for the $CsSnBr_3$ parent material synthesized by the solvent synthesis (SS), high temperature (HT) and mechanochemical synthesis (MCS) methods.	S15
**Figure S11.** UV-Vis absorption spectra (a) and Tauc plots showing direct bandgaps (b-d) for the $CsSnBr_3$ parent material synthesized by the solvent synthesis (SS), high temperature (HT) and mechanochemical synthesis (MCS) methods.	S15
**Figure S12.** Variable temperature (230-418 K) ^{119}Sn T_2* relaxation time as a function of absolute temperature for the $CsSnBr_3$ (SS) material.	S16
**Figure S13.** UV-Vis absorption spectra for $CsSnBr_3$ that was stored under ambient laboratory conditions over 300 days, pristine $CsSnBr_3$ and Cs_2SnBr_6.	S16
**Figure S14.** Solid-state ^{119}Sn NMR spectra of for degraded $CsSnBr_3$ parent and $SnBr_2$ starting precursor at 11.75 T acquired with spinning frequencies of 12 kHz with 2,000,000 scans each.	S17
**References**	S17

EXPERIMENTAL

Materials

Starting materials were purchased from the following commercial sources and were used without further modification: CsCl (Terochem Laboratories Ltd. 99.7%), CsBr (Sigma, 99.99%), $SnCl_2$ (Alfa Aesar, >99%), $SnBr_2$ (Alfa Aesar, 99.2%), $SnBr_4$ (Sigma, 99%), HBr (Anachemia, 48%), and H_3PO_2 (Sigma, 50 wt.% in H_2O).

High-Temperature Synthesis of $CsSn(Cl_{1-x}Br_x)_3$ ($0 \leq x \leq 1$): Various members of the solid solution $CsSn(Cl_{1-x}Br_x)_3$ were prepared by reactions at high temperature. CsX and SnX_2 (X = Cl, Br) were combined in stoichiometric ratios on a 0.5-g scale, finely ground using an agate mortar and pestle, pressed into pellets, and loaded into fused-silica tubes which were evacuated under a pressure of 10^{-3} mbar and sealed. The tubes were heated at 1.5 K/min to either 673 or 723 K (depending on composition), held at that temperature for 15 h, and then cooled to room temperature at 5 K/min. The samples were stored in glass vials and further characterized under ambient conditions.

Cubic $CsSnCl_3$ was obtained as a metastable phase at room temperature as follows: a sample of $CsSnCl_3$ prepared as described above was heated to 673 K, kept at this temperature for 15 h, cooled to 573 K at 5 K/min, and then quenched in an ice-water bath.

Mechanochemical Synthesis of $CsSnBr_3$: A mixture of 1.5 mmol each of CsBr and $SnBr_2$ was ground using an agate mortar and pestle for 10 min. Within an argon-filled glove box, the mixture was transferred to a 50-mL zirconia grinding vessel (containing *ca.* 50 g of zirconia balls with 3−8 mm diameter), which was sealed with parafilm to minimize exposure to air. The sample was ground in a Changsha Deco DECO-PBM-V-0.4L electric planetary ball mill at a rotation frequency of 550 rpm for 0.5 h. The vessel was opened to scratch its inner wall by using a clean spatula and sealed again under an inert atmosphere (Ar glove box). This process was repeated four times for a total of 2.5 h of grinding time.

Solvent Synthesis of $CsSnBr_3$: A mixture of 1 mmol each of CsBr and $SnBr_2$ was placed in a 40-mL glass vial to which 4.5 mL of concentrated HBr and 0.5 mL of H_3PO_2 were added. The mixture was heated under a nitrogen atmosphere to 120 °C on a hot plate, with continuous stirring by a magnetic stir bar. A black precipitate formed immediately. After the mixture was heated for 0.5 h, it was cooled over 1 h to room temperature. The precipitate was filtered under reduced pressure, washed with isopropyl alcohol, dried for 0.5 h, and quickly packed into a 4-mm o.d. ZrO_2 rotor for the solid-state NMR experiments.

Solvent Synthesis of Cs_2SnBr_6: A mixture of CsBr (2 mmol) and $SnBr_4$ (1 mmol) was placed in a 10-mL glass vial to which 5 mL of concentrated HBr was added. The mixture was heated to 120 °C on a hot plate, with continuous stirring by a magnetic stir bar. A white precipitate formed immediately. After the mixture was heated for 1 h, it was cooled over 1 h to room temperature. The precipitate was filtered using a Buchner funnel, washed with 95% ethanol, dried overnight, and stored in a vial under ambient conditions.

Powder X-ray Diffraction: Powder XRD patterns were collected on a Rigaku Ultima IV diffractometer equipped with a Co $K\alpha$ radiation source ($K_{\alpha1}$, 1.78900 Å; $K_{\alpha2}$, 1.79283 Å) operated at 38 kV and 38 mA, and a D/Tex Ultra detector with a Fe filter to eliminate K_β radiation (1.62083 Å). The samples were placed on zero background plates. Data were collected in continuous scan mode between 5 and 90° in 2θ with a step size of 0.0200°. Profile fitting was performed using the FullProf suite of software and unit cell parameters were refined.

Energy-dispersive X-ray Spectroscopy and Field Emission Scanning Electron Microscopy: Samples were examined on Zeiss Sigma 300 VP field emission scanning electron microscope equipped with dual silicon drift detectors for energy-dispersive X-ray spectroscopy to determine chemical compositions.

UV-Visible Diffuse Reflectance Spectroscopy: Diffuse reflectance spectra were collected on a Cary 5000 UV-vis-NIR spectrophotometer between 200 and 800 nm and calibrated with a Spectralon (>99%) reflectance standard. The diffuse reflectance spectra were converted to absorption spectra using the Kubelka-Munk function, $\alpha/S = (1-R)^2/2R$, where α is the Kubelka-Munk absorption coefficient, S is the scattering coefficient, and R is the reflectance. Direct bandgaps were extrapolated from the intercepts in Tauc plots of $(\alpha h\nu)^2$ vs E (eV).

Solid-State Nuclear Magnetic Resonance (NMR) Spectroscopy:

I. Cesium-133 NMR Spectroscopy: Solid-state ^{133}Cs NMR measurements were performed at 11.75 T (^{1}H, 500 MHz) on a Bruker Avance NEO 500 spectrometer, under magic angle spinning (MAS) conditions using a 4 mm H/X MAS Bruker probe with $\omega_o/2\pi(^{133}Cs)$ = 65.6 MHz. All samples were packed into 4 mm o.d. ZrO_2 rotors and NMR data were acquired using a Bloch decay pulse sequence using a short tip angle pulse of 1.38 μs (π/2 pulse = 5.5 μs, solution $\gamma B_1/2\pi$ = 45.5 kHz) with an acquisition time of 100 ms and an optimized recycle delay of 10 to 1800 s. The nuclear spin-lattice relaxation time (T_1) values of ^{133}Cs nuclei for all samples were measured using an inversion recovery pulse sequence. The T_1 values were calculated by fitting the peak intensity values using a three-parameter exponential decay function: $I_{(t)} = I_{(\infty)} + Ae^{-t/T_1}$, where $I_{(t)}$ and $I_{(\infty)}$ are the NMR signal intensities measured at time t and at t infinity, respectively, and T_1 are A are the spin-lattice relaxation time and pre-exponential constant, respectively. All ^{133}Cs NMR spectra discussed here were referenced by setting a 0.1 M $CsNO_3$ (aq.) solution at (^{133}Cs) = 0.00 ppm.

II. Tin-119 NMR Spectroscopy: Solid-state ^{119}Sn NMR measurements were performed at 11.75 T (^{1}H, 500 MHz) on a Bruker Avance NEO 500 spectrometer under both MAS and non-spinning conditions using a 4 mm H/X MAS Bruker probe with $\omega_o/2\pi(^{119}Sn)$ = 186.5 MHz. All samples were packed into 4 mm o.d. ZrO_2 rotors and NMR data were acquired using a 4.0 μs π/2 ($\gamma B_1/2\pi$ = 62.5 kHz) either with a Hahn-echo pulse sequence ($(\pi/2)_x - \tau_1 - (\pi)_y - \tau_2 -$ ACQ, where τ represents the interpulse and refocusing delays) or with a Bloch pulse sequence with an acquisition time of 4-5 ms and an optimized recycle delay of 0.01-200 s.

Variable temperature (VT) ^{119}Sn NMR spectra were acquired between 230 and 418 K with the same instrument under non-spinning conditions using a Bruker VT unit and calibrated using the ^{207}Pb chemical shifts of $MAPbCl_3$.[2] Dry N_2(g) was used as the VT gas and gas flow rates were adjusted to reach the target temperatures. The ^{119}Sn T_1 values were measured using an inversion recovery pulse sequence as discussed in the ^{133}Cs NMR experimental section. All ^{119}Sn NMR spectra were referenced by setting the ^{119}Sn signal of a tetracyclohexyl-tin(IV) powder to $\delta(^{119}Sn) = -97.35$ ppm, a secondary reference with respect to $Sn(CH_3)_4$ at $\delta(^{119}Sn) = 0.00$ ppm.

Table S1. Elemental analysis of $CsSn(Cl_{1-x}Br_x)_3$ materials measured by EDS.

Sample (Nominal composition)	Elemental atomic %				%Cl : %Br
	Cs	Sn	Cl	Br	
$CsSnCl_3$	19.3	19.4	61.3	-	100 : 0
$CsSn(Cl_{0.90}Br_{0.10})_3$	18.9	19.5	54.6	7.0	89 : 11
$CsSn(Cl_{0.67}Br_{0.33})_3$	18.8	19.4	39.6	22.2	64 : 36
$CsSn(Cl_{0.50}Br_{0.50})_3$	18.8	18.6	29.4	33.2	47 : 53
$CsSn(Cl_{0.33}Br_{0.67})_3$	18.3	19.1	19.0	43.6	30 : 70
$CsSn(Cl_{0.10}Br_{0.90})_3$	19.4	18.7	6.0	55.9	10 : 90
$CsSnBr_3$	17.3	18.0	-	64.7	0 : 100

Table S2. Unit cell constants (a) and direct bandgap values for $CsSn(Cl_{1-x}Br_x)_3$ materials.

Sample	Unit cell constant, a (Å)	Direct bandgap (eV)
$CsSnCl_3$ (cubic)	5.5894(5)	2.83
$CsSn(Cl_{0.90}Br_{0.10})_3$	5.6108(3)	2.71
$CsSn(Cl_{0.67}Br_{0.33})_3$	5.6642(3)	2.42
$CsSn(Cl_{0.50}Br_{0.50})_3$	5.6991(6)	2.26
$CsSn(Cl_{0.33}Br_{0.67})_3$	5.7353(7)	2.10
$CsSn(Cl_{0.10}Br_{0.90})_3$	5.7830(2)	1.87
$CsSnBr_3$	5.8031(3)	1.79

Table S3. Room temperature solid-state ^{133}Cs NMR experimental parameters, chemical shifts, fwhm and spin-lattice relaxation (T_1) values for $CsSn(Cl_{1-x}Br_x)_3$ materials. The ^{133}Cs NMR spectra were acquired at 11.75 T using a Bloch pulse sequence.

Sample	ν_{rot} (kHz)	Recycle delay (s)	^{133}Cs fwhm (Hz) ± 5	$\delta(^{133}Cs)$ (ppm) ± 0.1	$T_1(^{133}Cs)$ (s)
$CsSnCl_3$ (cubic)	13.0	300	138	64.7	14.8 ± 0.2
$CsSn(Cl_{0.90}Br_{0.10})_3$		60	92	68.6	9.8 ± 0.2
$CsSn(Cl_{0.67}Br_{0.33})_3$		100	82	73.7	15.3 ± 0.4
$CsSn(Cl_{0.50}Br_{0.50})_3$		60	114	74.7	19.7 ± 0.1
$CsSn(Cl_{0.33}Br_{0.67})_3$		60	149	73.4	25.0 ± 0.1
$CsSn(Cl_{0.10}Br_{0.90})_3$		60	125	67.7	35.3 ± 0.1
$CsSnBr_3$		60	70	64.0	34.9 ± 0.1

Table S4. Room temperature solid-state ^{119}Sn NMR experimental parameters, chemical shifts, fwhm and spin-lattice relaxation (T_1) values for $CsSn(Cl_{1-x}Br_x)_3$ materials. The ^{119}Sn NMR spectra were acquired at 11.75 T using a Hahn-echo (ν_{rf} = 62.5 kHz).

Sample	ν_{rot} (kHz)	Recycle delay (s)	$\delta(^{119}Sn)$ (ppm)	^{119}Sn fwhm (kHz) ± 0.1	$T_1(^{119}Sn)$ (s)
$CsSnCl_3$ (cubic)	10.0	10	−562 ± 1	1.8	3.5
$CsSn(Cl_{0.90}Br_{0.10})_3$		1	−544 ± 1	1.4	3.4
$CsSn(Cl_{0.67}Br_{0.33})_3$		1	−510 ± 1	1.7	2.4
$CsSn(Cl_{0.50}Br_{0.50})_3$		5	−487 ± 1	2.7	3.0×10^{-1}
$CsSn(Cl_{0.33}Br_{0.67})_3$		1	−458 ± 2	4.3	2.7×10^{-2}
$CsSn(Cl_{0.10}Br_{0.90})_3$		0.01	−365 ± 5	17.0	8.0×10^{-3}
$CsSnBr_3$		0.20	−289 ± 5	19.0	5.6×10^{-3}

Table S5. Room temperature solid-state ^{119}Sn NMR experimental parameters, chemical shifts, fwhm and spin-lattice relaxation (T_1) for $CsSnBr_3$ materials prepared by the solvent synthesis (SS), high temperature (HT) and mechanochemical synthesis (MCS) methods. The ^{119}Sn NMR spectra were acquired at 11.75 T using a Hahn-echo (ν_{rf} = 62.5 kHz).

Sample	ν_{rot} (kHz)	Recycle delay (s)	$\delta(^{119}Sn)$ (ppm)	^{119}Sn fwhm (kHz)	$T_1(^{119}Sn)$ (s)
$CsSnBr_3$ (SS)	0.0	0.05	−386 ± 2	7.5 ± 0.2	0.025 ± 0.001
$CsSnBr_3$ (HT)		0.2	−284 ± 5	21.0 ± 0.5	0.007 ± 0.001
$CsSnBr_3$ (MCS)		0.2	−295 ± 10	34.0 ± 1.0	0.003 ± 0.001

Table S6. Variable-temperature ^{119}Sn NMR parameters for $CsSnBr_3$ (SS) acquired under non-spinning sample conditions at 11.75 T using a 4.0 µs π/2 Bloch pulse sequence.

T(set) (K)	T(calibrated) (±2) (K)	$\delta_{iso}(^{119}Sn)$ † (ppm)	Ω (ppm) †	*κ* †	^{119}Sn *fwhm (kHz)* ± 0.1	^{119}Sn T_2* *(ms)*	^{119}Sn T_1 (ms)
233	229.5	−393	85	−0.1	14.7	0.068	635(200)
253	250.9	−393	75	−0.1	13.6	0.074	390(85)
273	273.6	−390	75	−0.25	10.6	0.094	123(12)
292.4	295.4	−386	35	−1.0	7.2	0.139	25(1)
313	318.7	−371	0	NA	6.5	0.154	14(1)
333	341.5	−364	0	NA	6.1	0.164	8.8(5)
353	366.4	−350	0	NA	5.7	0.175	4.12(15)
373	385.5	−334	0	NA	6.0	0.167	2.16(9)
393	407.6	−319.6	0	NA	5.4	0.185	1.14(5)
403	417.9	−317.7	0	NA	4.5	0.222	0.92(2)

NA – not applicable; † - δ_{iso}, Ω and κ are isotropic chemical shift, span and skew, respectively,

where $\delta_{iso} = \frac{\delta_{11} + \delta_{22} + \delta_{33}}{3}$, $\Omega = (\delta_{11} - \delta_{33})$ *and* $\kappa = 3(\frac{\delta_{22} - \delta_{iso}}{\Omega})$

Supplementary note 1:

The variable temperature ^{119}Sn spin-lattice relaxation time (T_1) is related to the absolute temperature values for $CsSnBr_3$ (SS) as shown in Table S6. $\log_{10}[T_1/s]$ linearly depends on [1000/T(K)] (i.e., an Arrhenius relationship) within the temperature range of 230 to 418 K. The slope of the Arrhenius fit is related to the activation energy as $E_a = (2303 \cdot R) \cdot (slope)$, where R = 8.314 J/(mol·K). A slope value of 1.51 ± 0.06 K was obtained from the least-squares Arrhenius fit; hence E_a = 28.9 ± 1.2 kJ/mol or 0.30 ± 0.01 eV

Table S7. Solid-state ^{133}Cs NMR acquisition parameters and experimental conditions used for the $CsSnBr_3$ areal degradation study (see Figure 9b in the manuscript). The ^{133}Cs NMR spectra were acquired at 11.75 T using a Bloch pulse sequence.

Material	T_1 (s)	ν_{rot} (kHz)	Recycle delay (s)	# of scans	Acquisition time (min)
$CsSnBr_3$ (degraded)	-		1800	4	120
$CsSnBr_3$	34.9	5 kHz	60	4	4
Cs_2SnBr_6	170		500	4	33
CsBr	631		300	4	20

Table S8. Solid-state ^{119}Sn NMR acquisition parameters and experimental conditions used for the $CsSnBr_3$ areal degradation study (see Figure 9c in the manuscript). The ^{119}Sn NMR spectra were acquired at 11.75 T using a Hahn-echo (ν_{rf} = 62.5 kHz).

Material		T_1 (s)	ν_{rot} (kHz)	Recycle delay (s)	# of scans	Acquisition time (min)
$CsSnBr_3$ (degraded)	region-1	-	0	0.2	512	1.7
	region-1		10	0.2	512	1.7
	region-2	-	10	100	512	853
	region-3	-	10	100	512	853
	region-4	-	12	0.01	2,000,000	736
$CsSnBr_3$		0.006	10	0.2	1024	3.4
Cs_2SnBr_6		12.3	10	100	32	53
SnO_2		3.1[ref.3]	10	30	32	15
$SnBr_4$		1.95[ref.4]	0	3	512	25.6
$SnBr_2$ (trace metal grade)	$SnBr_2$ region	1.12[ref.4]	13	1.5	512	12.8
	β-Sn region	0.00012 [ref.4]	12	0.005	2,000,000	537

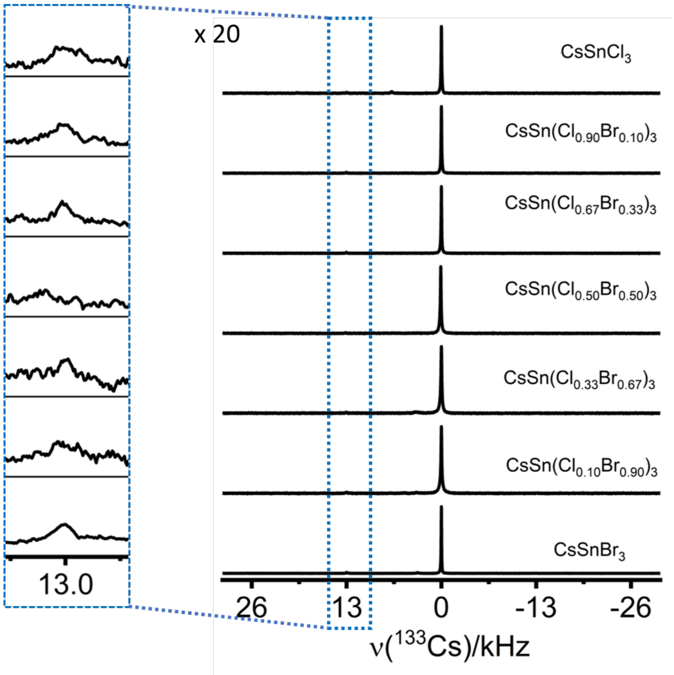

Figure S1. *Room temperature solid-state ^{133}Cs NMR spectra of CsSn($Cl_{1-x}Br_x$)$_3$ materials at 11.75 T with a magic angle spinning frequency of 13 kHz. Expansion shows the corresponding spinning side band.*

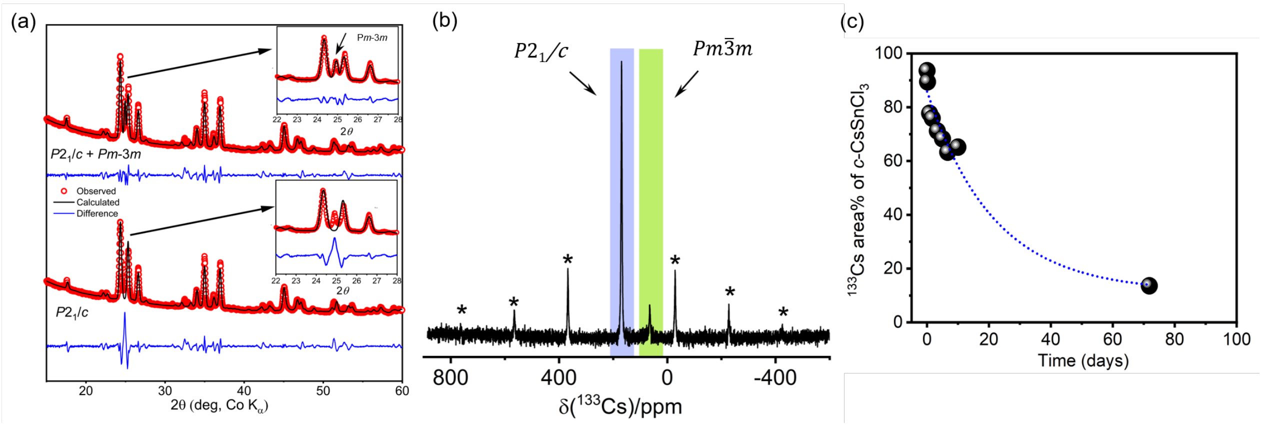

Figure S2. *Room temperature PXRD (a) and ^{133}Cs NMR spectra (b) of $CsSnCl_3$ materials prepared by the high-temperature sealed-tube method followed by slow-cooling (5 K/min). PXRD patterns were collected within 24 h of synthesis and ^{133}Cs NMR spectra were acquired at 11.75 T after six days of synthesis. The asterisks (*) in (b) indicate spinning sidebands for monoclinic $CsSnCl_3$. Plot of ^{133}Cs NMR peak area for the cubic $CsSnCl_3$ phase as a function of time (up to 72 days) showing mono-exponential like decay kinetics for the cubic to monoclinic phase transition in $CsSnCl_3$ (c). The data points between 10 to 72 days are missing because of the sudden institutional lockdown (~ 60 days) during the first wave of COVID-19 pandemic in early 2020.*

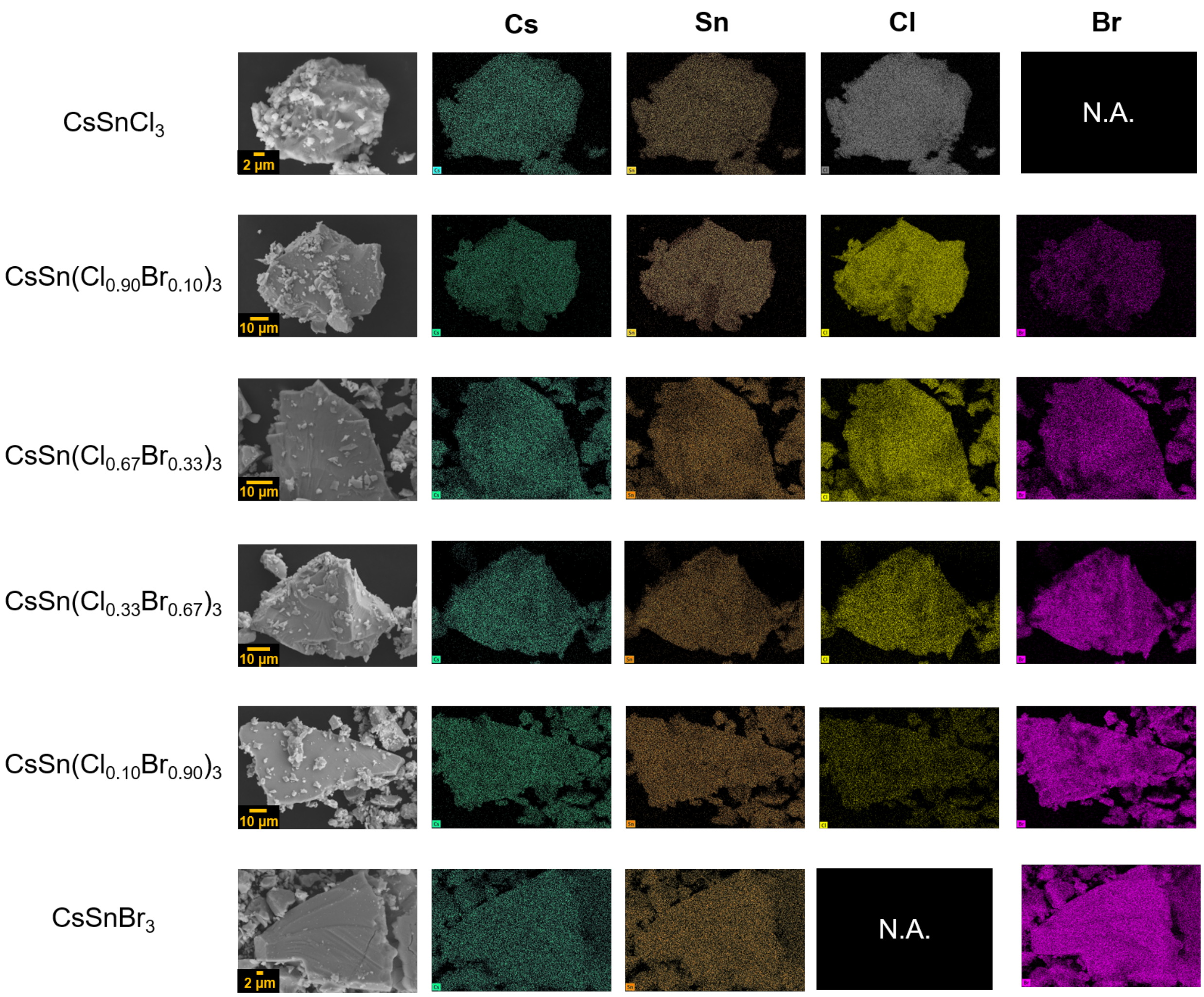

Figure S3. *FESEM images and the corresponding EDS elemental mapping for Cs, Sn, Cl and Br for the* $CsSn(Cl_{1-x}Br_x)_3$ *materials (top to bottom: x = 0.00, 0.10, 0.33, 0.67 and 1.00).*

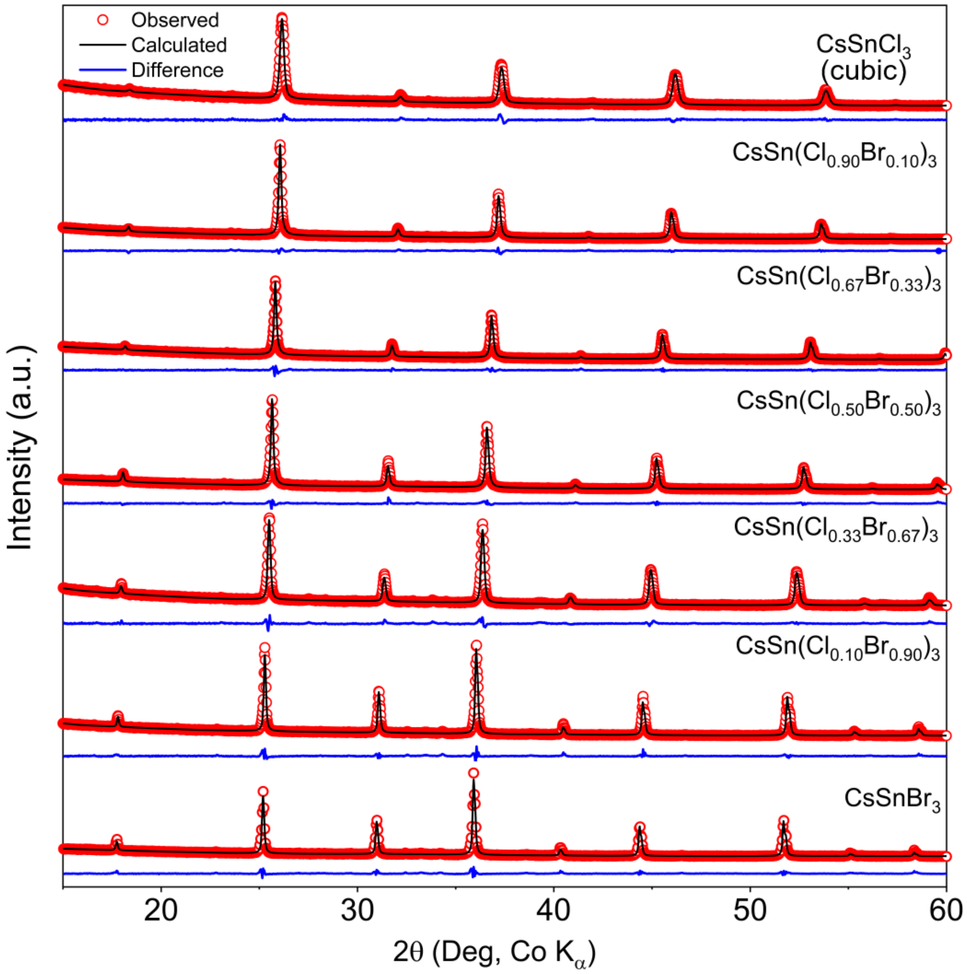

Figure S4. *Room temperature experimental and fitted PXRD diagrams for the* $CsSn(Cl_{1-x}Br_x)_3$ *series. All the diffraction data are fitted assuming a cubic (Pm-3m) space group symmetry.*

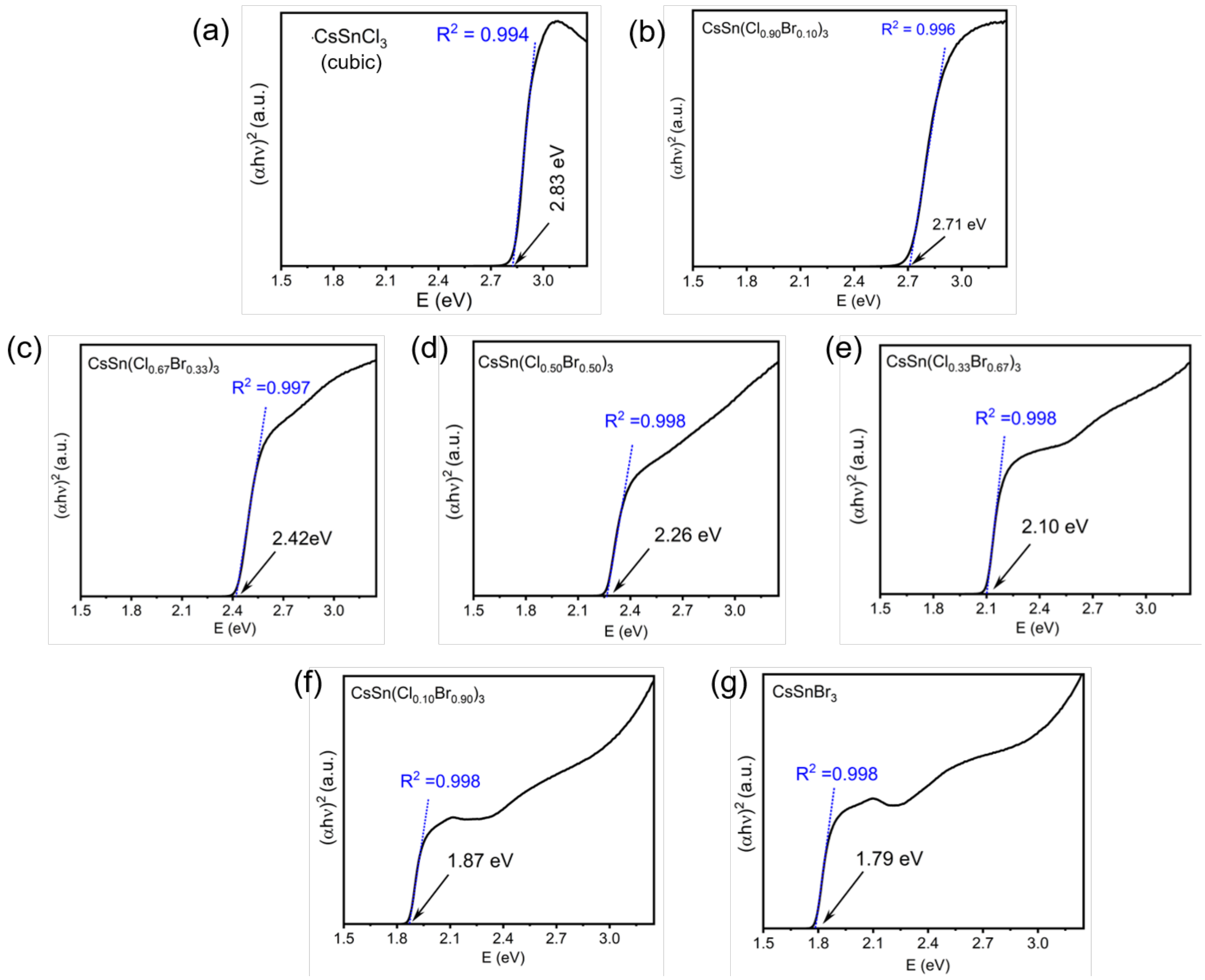

Figure S5. *Tauc plots showing direct bandgaps of the cubic phases of* $CsSn(Cl_{1-x}Br_x)_3$ *materials; c-*$CsSnCl_3$ *(a),* $CsSn(Cl_{0.90}Br_{0.10})_3$ *(b),* $CsSn(Cl_{0.67}Br_{0.33})_3$ *(c),* $CsSn(Cl_{0.50}Br_{0.50})_3$ *(d),* $CsSn(Cl_{0.33}Br_{0.67})_3$ *(e),* $CsSn(Cl_{0.10}Br_{0.90})_3$ *(f) and* $CsSnBr_3$ *(g).*

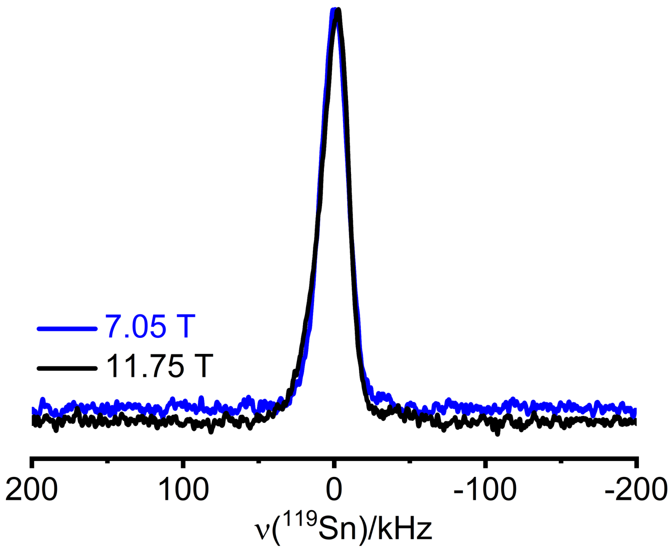

Figure S6. *Solid-state* ^{119}*Sn NMR spectra of* $CsSnBr_3$ *at 7.05 and 11.75 T under non-spinning sample conditions. The scales are vertically normalized.*

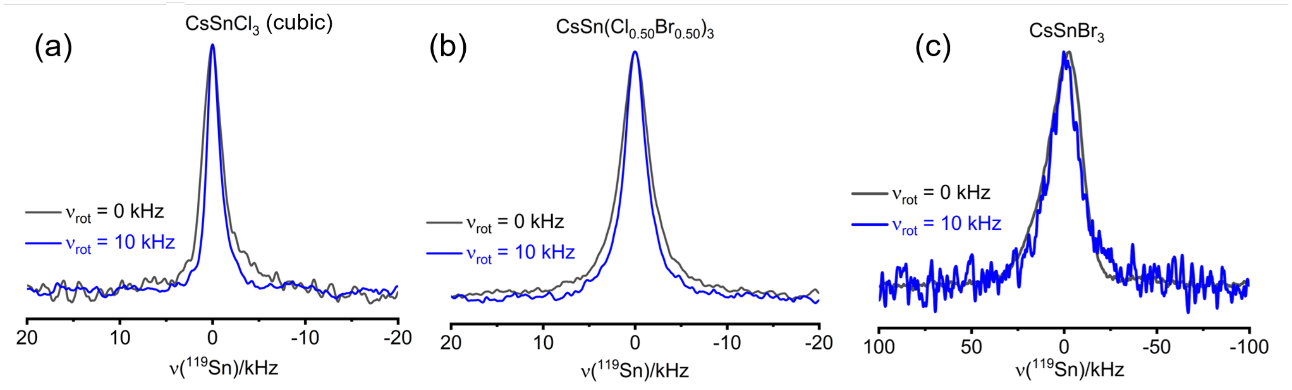

Figure S7. *Solid-state* ^{119}*Sn NMR spectra of c-*$CsSnCl_3$ *(a),* $CsSn(Cl_{0.50}Br_{0.50})_3$ *(b) and* $CsSnBr_3$ *(c) at 11.75 T acquired with spinning frequencies of 0 and 10 kHz. The scales are vertically normalized.*

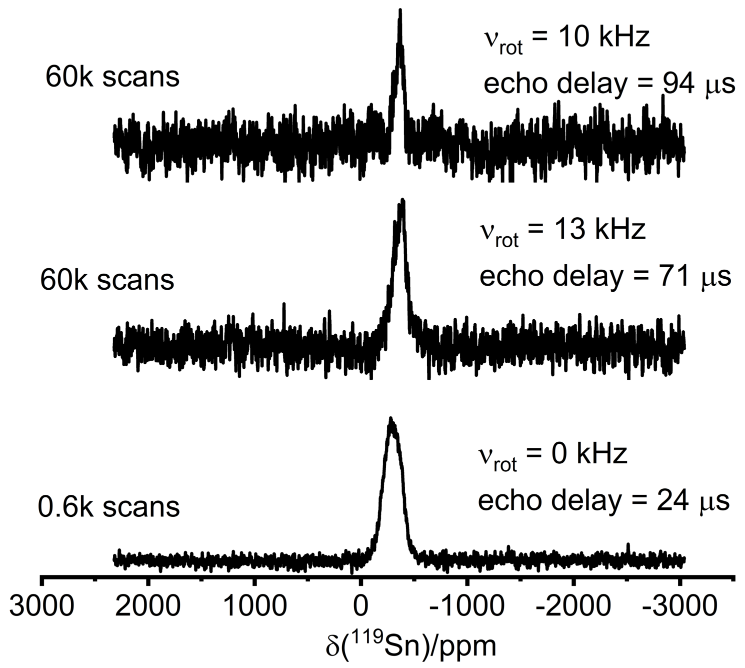

Figure S8. *Solid-state ^{119}Sn NMR spectra of $CsSn(Cl_{0.10}Br_{0.90})_3$ at 11.75 T acquired with spinning frequencies between 0 to 13 kHz with the Hahn-echo pulse sequence and with various echo-delays as indicated. The scales are vertically normalized.*

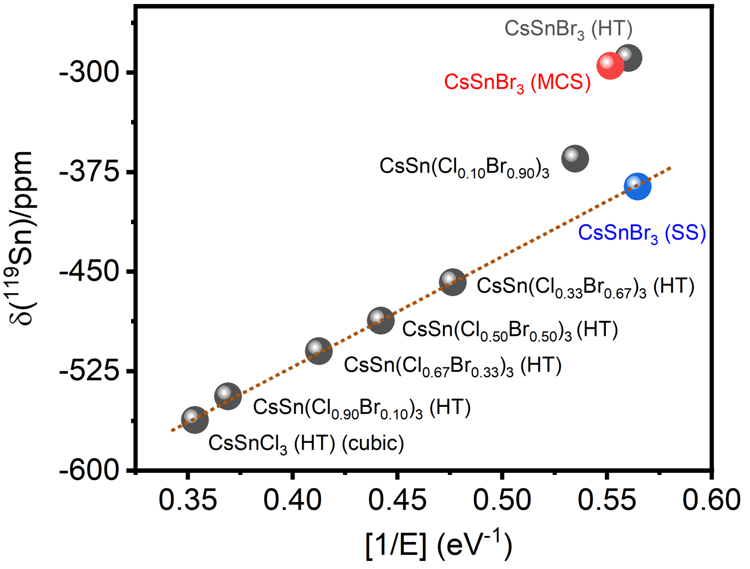

Figure S9. *Solid-state ^{119}Sn NMR chemical shifts vs the inverse of direct bandgap values for the $CsSn(Cl_{1-x}Br_x)_3$ series.*

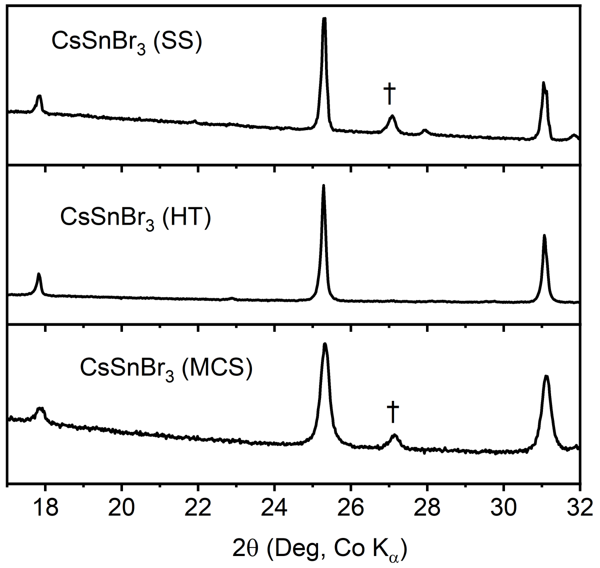

Figure S10. *Room temperature PXRD patterns for the $CsSnBr_3$ parent material synthesized by the solvent synthesis (SS), high temperature (HT) and mechanochemical synthesis (MCS) methods. The dagger (†) indicates signal from Cs_2SnBr_6.*

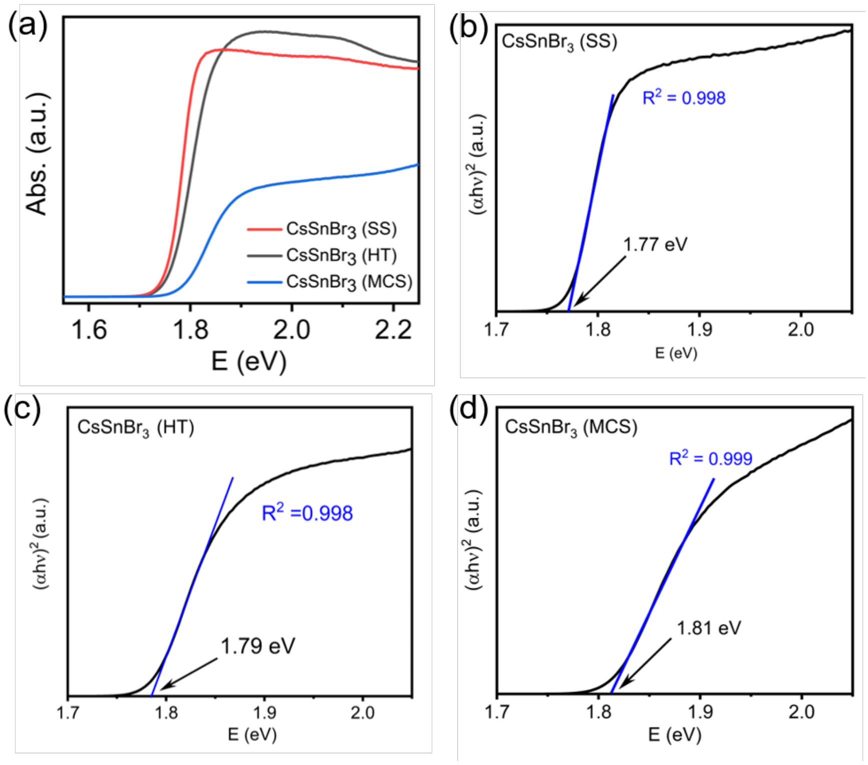

Figure S11. *UV-Vis absorption spectra (a) and Tauc plots showing direct bandgaps (b-d) for the $CsSnBr_3$ parent material synthesized by the solvent synthesis (SS), high temperature (HT) and mechanochemical synthesis (MCS) methods.*

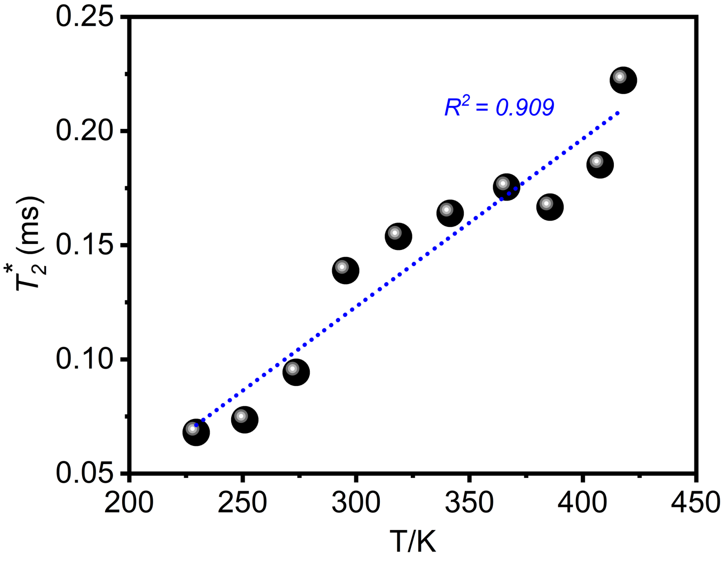

Figure S12. *^{119}Sn T_2* (i.e., $\frac{1}{fwhm}$) relaxation time as a function of absolute temperature (230-418 K) for the $CsSnBr_3$ (SS) material. ^{119}Sn NMR spectra were acquired at 11.75 T under non-spinning sample conditions. The data were fit by a least-squares method with the following equation: $T_2^*/ms = -0.09756(0.02587) + 7.35516(0.77322) \times 10^{-4} \cdot T/K$*

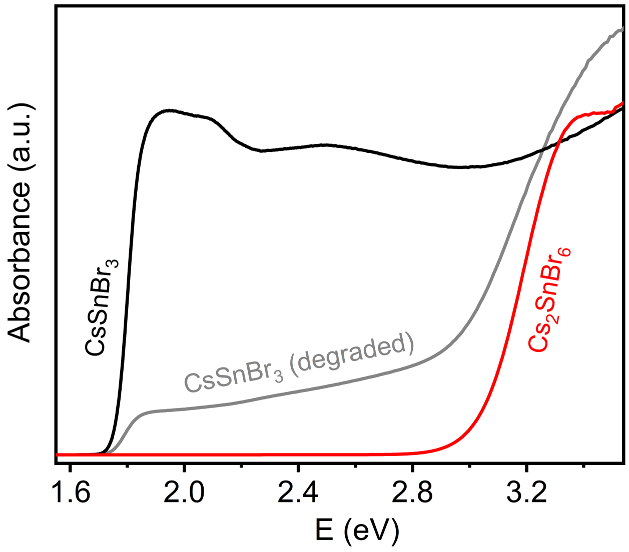

Figure S13. *UV-Vis absorption spectra for a $CsSnBr_3$ sample that was stored under ambient laboratory conditions over 300 days (degraded $CsSnBr_3$), pristine $CsSnBr_3$ and Cs_2SnBr_6.*

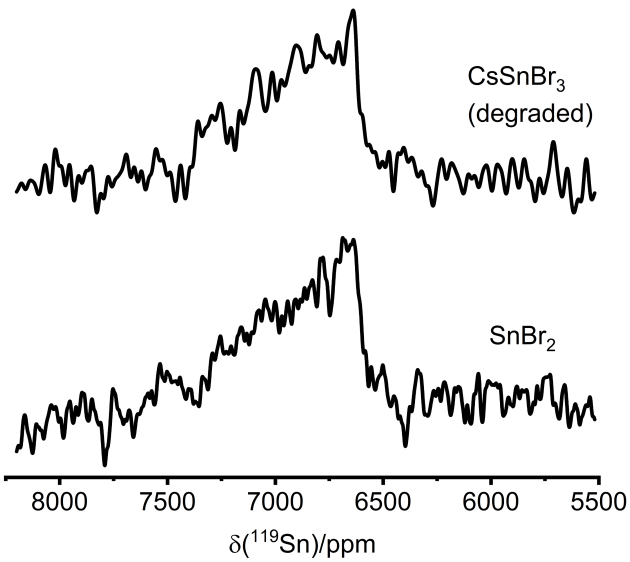

***Figure S14**. Solid-state ^{119}Sn NMR spectra of the degraded $CsSnBr_3$ parent and $SnBr_2$ starting precursor at 11.75 T acquired with spinning frequencies of 12 kHz with 2,000,000 scans each.*

References:

(1) Kubelka, P.; Munk, F. Ein Beitrag Zur Optik Der Farbanstriche. *Z.Tech. Phys. (Leipzig)* **1931**, *12*, 593–601.

(2) Bernard, G. M.; Goyal, A.; Miskolzie, M.; McKay, R.; Wu, Q.; Wasylishen, R. E.; Michaelis, V. K. Methylammonium Lead Chloride: A Sensitive Sample for an Accurate NMR Thermometer. *J. Magn. Reson.* **2017**, *283*, 14–21.

(3) Cossement, C.; Darville, J.; Gilles, J. -M; Nagy, J. B.; Fernandez, C.; Amoureux, J. -P. Chemical Shift Anisotropy and Indirect Coupling in SnO_2 and SnO. *Magn. Reson. Chem.* **1992**, *30* (3), 263–270.

(4) Kubicki, D. J.; Prochowicz, D.; Salager, E.; Rakhmatullin, A.; Grey, C. P.; Emsley, L.; Stranks, S. D. Local Structure and Dynamics in Methylammonium, Formamidinium and Cesium Tin(II) Mixed−halide Perovskites from ^{119}Sn Solid−State NMR. *J. Am. Chem. Soc.* **2020**, *142*, 7813–7826.
